# Aberrant miR-29 is a predictive feature of severe phenotypes in pediatric Crohn’s disease

**DOI:** 10.1172/jci.insight.168800

**Published:** 2024-02-22

**Authors:** Alexandria J. Shumway, Michael T. Shanahan, Emilie Hollville, Kevin Chen, Caroline Beasley, Jonathan W. Villanueva, Sara Albert, Grace Lian, Moises R. Cure, Matthew Schaner, Lee-Ching Zhu, Surekha Bantumilli, Mohanish Deshmukh, Terrence S. Furey, Shehzad Z. Sheikh, Praveen Sethupathy

**Affiliations:** 1 Department of Biomedical Sciences, Cornell University, Ithaca, New York, USA.; 2 Neuroscience Center,; 3 Center for Gastrointestinal Biology and Disease,; 4 Department of Genetics,; 5 Department of Pathology and Laboratory Medicine, and; 6 Department of Biology, The University of North Carolina at Chapel Hill, Chapel Hill, North Carolina, USA.

**Keywords:** Gastroenterology, Genetics, Inflammatory bowel disease

## Abstract

Crohn’s disease (CD) is a chronic inflammatory gut disorder. Molecular mechanisms underlying the clinical heterogeneity of CD remain poorly understood. MicroRNAs (miRNAs) are important regulators of gut physiology, and several have been implicated in the pathogenesis of adult CD. However, there is a dearth of large-scale miRNA studies for pediatric CD. We hypothesized that specific miRNAs uniquely mark pediatric CD. We performed small RNA-Seq of patient-matched colon and ileum biopsies from treatment-naive pediatric patients with CD (*n* = 169) and a control cohort (*n* = 108). Comprehensive miRNA analysis revealed 58 miRNAs altered in pediatric CD. Notably, multinomial logistic regression analysis revealed that index levels of ileal miR-29 are strongly predictive of severe inflammation and stricturing. Transcriptomic analyses of transgenic mice overexpressing miR-29 show a significant reduction of the tight junction protein gene Pmp22 and classic Paneth cell markers. The dramatic loss of Paneth cells was confirmed by histologic assays. Moreover, we found that pediatric patients with CD with elevated miR-29 exhibit significantly lower Paneth cell counts, increased inflammation scores, and reduced levels of PMP22. These findings strongly indicate that miR-29 upregulation is a distinguishing feature of pediatric CD, highly predictive of severe phenotypes, and associated with inflammation and Paneth cell loss.

## Abstract 2

<p>Upregulation of miR-29b is highly predictive of inflammation severity and stricturing in human pediatric Crohn&rsquo;s disease and drives paneth cell loss in mouse models.</p>

## Graphical abstract

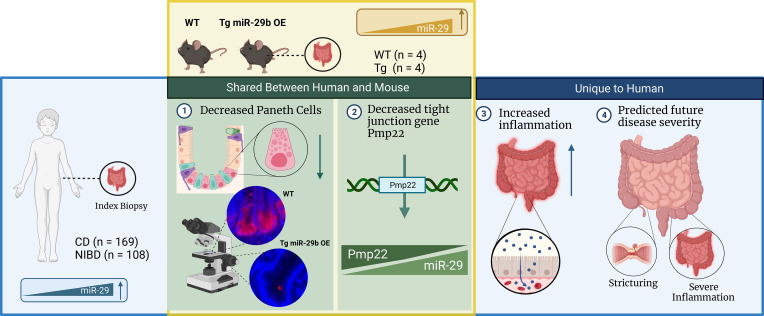

## Introduction

Crohn’s disease (CD) is a primary inflammatory bowel disease (IBD) and is thought to develop due to dysregulated inflammatory responses in genetically susceptible individuals. Over the course of the past decade especially, CD has become an increasingly global disease, with growing incidence in newly industrialized countries (1–3). The number of CD cases is predicted to increase in the United States by almost 1.5-fold by 2025 (4). Some reports have attributed this growth in substantive part to the increase in CD cases among the pediatric population; incidence of these types of cases has more than doubled since the start of the 21st century, and the pediatric population remains the fastest-growing affected age group (5–8).

The precise causes of CD are still enigmatic, but CD is believed to be an aberrant immune response to a complex interaction of factors including environmental exposures, genetics, and the gut microbiome. Both adult and pediatric CD are characterized by noncontiguous lesions in the gastrointestinal tract that can lead to chronic abdominal pain, diarrhea, fistulas, and/or abscesses. Up to 30% of patients with CD are pediatric, and these patients tend to exhibit a more severe phenotype due to concomitant issues of growth failure, poor bone density, and delayed puberty (9, 10). Although for some patients the existing therapies can aid in mucosal healing to decrease the need for surgical intervention and improve the overall quality of life, there is currently no cure to CD (10, 11). Treatment of CD can differ greatly across patients, since the appropriate therapeutic regimen relies on many factors including location and behavior of disease, comorbidities, previous treatments, and age (12, 13). Remission of CD remains difficult to achieve, especially in pediatric patients (12). The complexity and heterogeneity of CD and the highly variable efficacy of existing therapeutic options highlight the need for novel intervention methods. The development of novel biomarkers and prognostic indicators could further aid clinicians in determining disease trajectory and response to therapy (14).

In recent years, we and others have investigated microRNAs (miRNAs) as potential diagnostic markers, prognostic indicators of disease severity, and candidate therapeutic targets for adult CD (15–17). miRNAs are small noncoding RNAs (~22 nts long) that posttranscriptionally regulate gene expression and have been shown to influence most major biological processes and diseases (18). These molecules regulate the majority of protein-coding genes, and each miRNA can target up to hundreds of mRNAs, resulting in mRNA destabilization or inhibition of translation (18, 19). Dysfunction of miRNA activity can lead to acute or chronic inflammation, which is characteristic of CD (20, 21). In 2010, a seminal study showed the contribution of overall miRNA activity to intestinal architecture and function (20). More recently, specific miRNAs have been implicated in IBD (22–24). For example, we showed that a single miRNA (miR-31) is a major driver of the differences between 2 molecular subtypes of adult CD (25). Further functional studies demonstrate that miR-31 regulates barrier function, in part, by controlling the expression of activin A receptor-like type 1 (ALK1) and that high levels of miR-31 are strongly associated with poor clinical outcomes and increased likelihood of relapse after remission (26). This study, as well as several others, have established miRNAs as valuable prognostic indicators of disease behavior and potential therapeutic targets in adult CD (15, 24, 27).

Despite these advances, a major limitation of these studies is that they pertain largely to adult CD. There are only a handful of studies that focus on miRNAs in pediatric CD, and most of these do not use a sequencing-based approach to define comprehensive miRNA profiles (24, 27–33). Recently, we performed large-scale sequencing of miRNAs in pediatric CD, but we focused the analysis on only 1 miRNA (25). Here, we substantially expanded the cohort and performed a comprehensive quantitative analysis of all miRNAs in matched ileum and colon samples from pediatric patients and patients without IBD (non-IBD [NIBD]) as controls. We discovered and report on specific miRNAs that exhibit the greatest utility as predictive molecular features of key clinical outcomes in pediatric CD. We also performed functional follow-up studies in vivo of 1 particular miRNA that is a distinguishing feature of pediatric CD (relative to adult CD) and define potential targets and functions that merit further investigation.

## Results

### Ileal and colonic miRNA profiles stratify pediatric CD from patients with NIBD.

**Figure 1:**
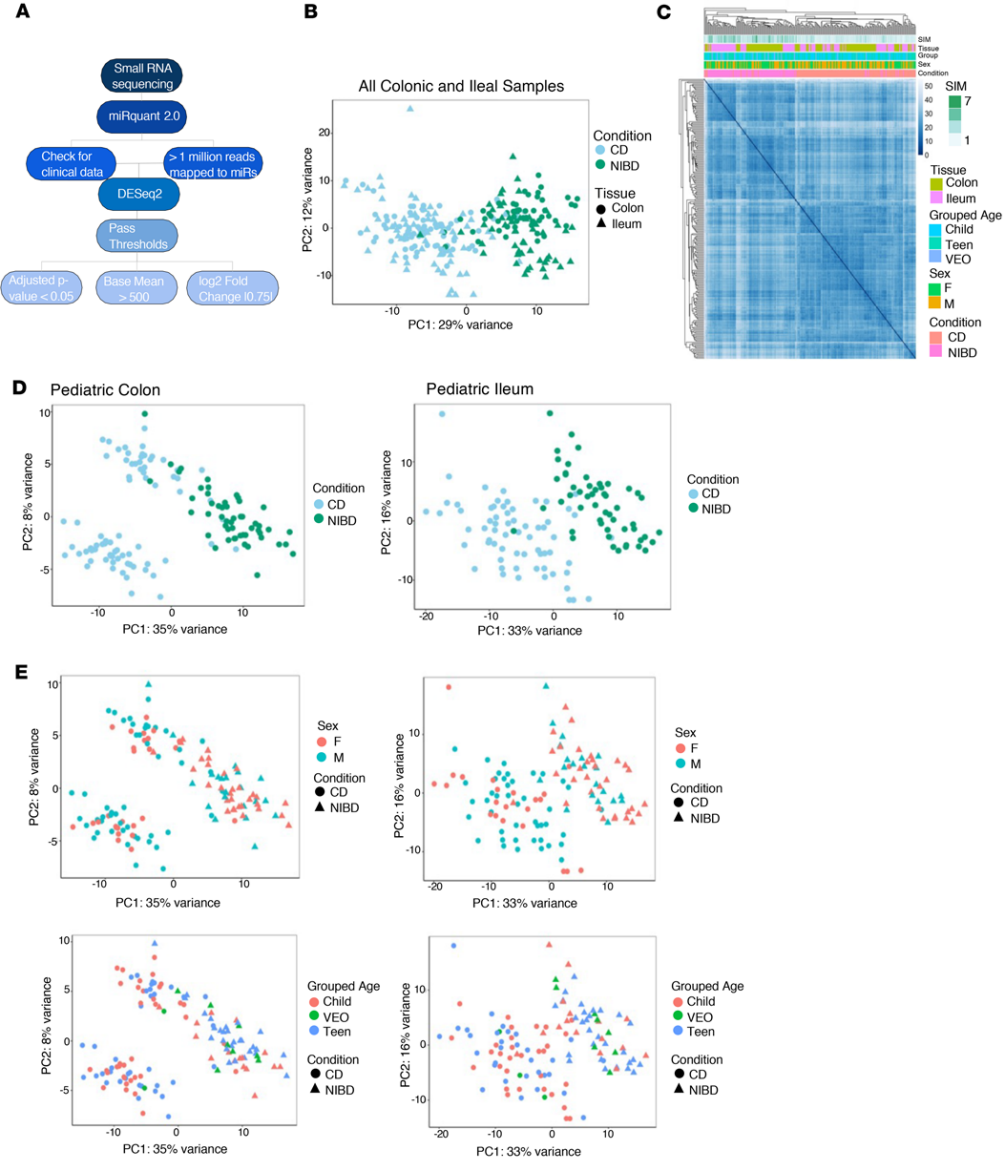
Ileal and colonic microRNA profiles stratify by disease status. (**A**) Workflow for smRNA-Seq analysis. (**B**) Principal component analysis (PCA) of variance stabilizing transformation (VST) normalized counts for all (*n* = 245) CD and NIBD samples accounting for the covariates of small RNA integrity metric (SIM), grouped ages (VEO ≤ 6; child = 7–12; teen = 13–17), and sex. The samples are colored in blue and green for CD and NIBD, respectively. The 2 tissue types are represented as circles for colon samples and triangles for ileal samples. The percent of variation explained is indicated for principal component 1 along the *x* axis and principal component 2 along the *y* axis. (**C**) Unsupervised hierarchical clustering of the Euclidean distances among all (*n* = 245) pediatric samples was calculated based on VST normalized counts accounting for the covariates of SIM, grouped ages, and sex. The CD and NIBD samples are indicated by peach and pink boxes, respectively. Other covariates are represented as the colors indicated by the legend. (**D**) PCA of pediatric miRNA profiles in colonic (*n* = 127) (left) and ileal (*n* = 118) (right) tissue accounting for the covariates of SIM, grouped ages, and sex. (**E**) PCA plots for colon (left) and ileum (right) in which the grouped patient age and sex phenotype information are overlaid. The colors green, red, and blue represent the VEO, child, and teen grouped ages, respectively. Female and male patients are indicated by red and blue, respectively. Disease status is specified by shape.

We had previously performed small RNA-Seq (smRNA-Seq) on 60 ileum and 76 colon tissue index biopsies from pediatric patients with CD as well as 50 ileum and 48 colon tissue samples from NIBD individuals (25). In that study, we focused our analysis on only 1 miRNA of interest. Therefore, the full potential of the predictive power of miRNAs for clinical outcomes in pediatric CD remained unknown. To fill this important knowledge gap, and to define a comprehensive miRNA signature of pediatric CD, we first expanded the cohort to 277 total samples and then implemented and applied the bioinformatic analysis pipeline miRquant 2.0. Subsequent to the implementation of this pipeline, we removed data sets with less than 1 million reads mapped to miRNAs (Figure 1A and Table 1). We also removed from further analysis those samples from individuals without detailed clinical information, including smRNA integrity metric (SIM), age, and sex. The demographics of the pediatric patients whose samples remained for further analysis are provided in Table 2 (*n* = 245).

Unsupervised hierarchical clustering and principal component analysis (PCA) of the miRNA profiles across all 245 samples showed a strong stratification by disease status (CD versus NIBD) (Figure 1, B and C). This result was maintained when considering only the patient-matched samples (*n* = 228) (Supplemental Figure 1A; supplemental material available online with this article; https://doi.org/10.1172/jci.insight.168800DS1). PCA of the colonic and ileal miRNA data sets, separately, also revealed a robust grouping of samples by disease (CD versus NIBD) along the first principal component (Figure 1D). There was no additional stratification detected according to age, sex, race, or ethnicity (Figure 1E and Supplemental Figure 1B).

### Colonic miRNAs separate pediatric CD into 2 clusters.

Performing PCA analysis on each tissue individually, pediatric patients with CD appear to be stratified into 2 clusters based on colonic miRNA profiles (Figure 1D), while this is not clearly evident with ileal miRNA profiles (Figure 1D). Intriguingly, it is apparent that the clustering among colonic CD cases is partially due to future ileal stricturing status (Supplemental Figure 2A). To define the colonic miRNAs that may explain this stratification, we sought to identify the miRNAs that are significantly differentially expressed between the 2 clusters (Supplemental Figure 2B). Using DESeq2, we found 2 colonic miRNAs (miR-99b and miR-146b) significantly more highly expressed in cluster #1, which includes all but 1 of the individuals who developed ileal stricturing, and found 1 colonic miRNA (miR-451a) significantly more highly expressed in cluster #2 (Supplemental Figure 2C).

### Many but not all miRNAs significantly altered in pediatric CD are shared between ileum and colon.

**Figure 2:**
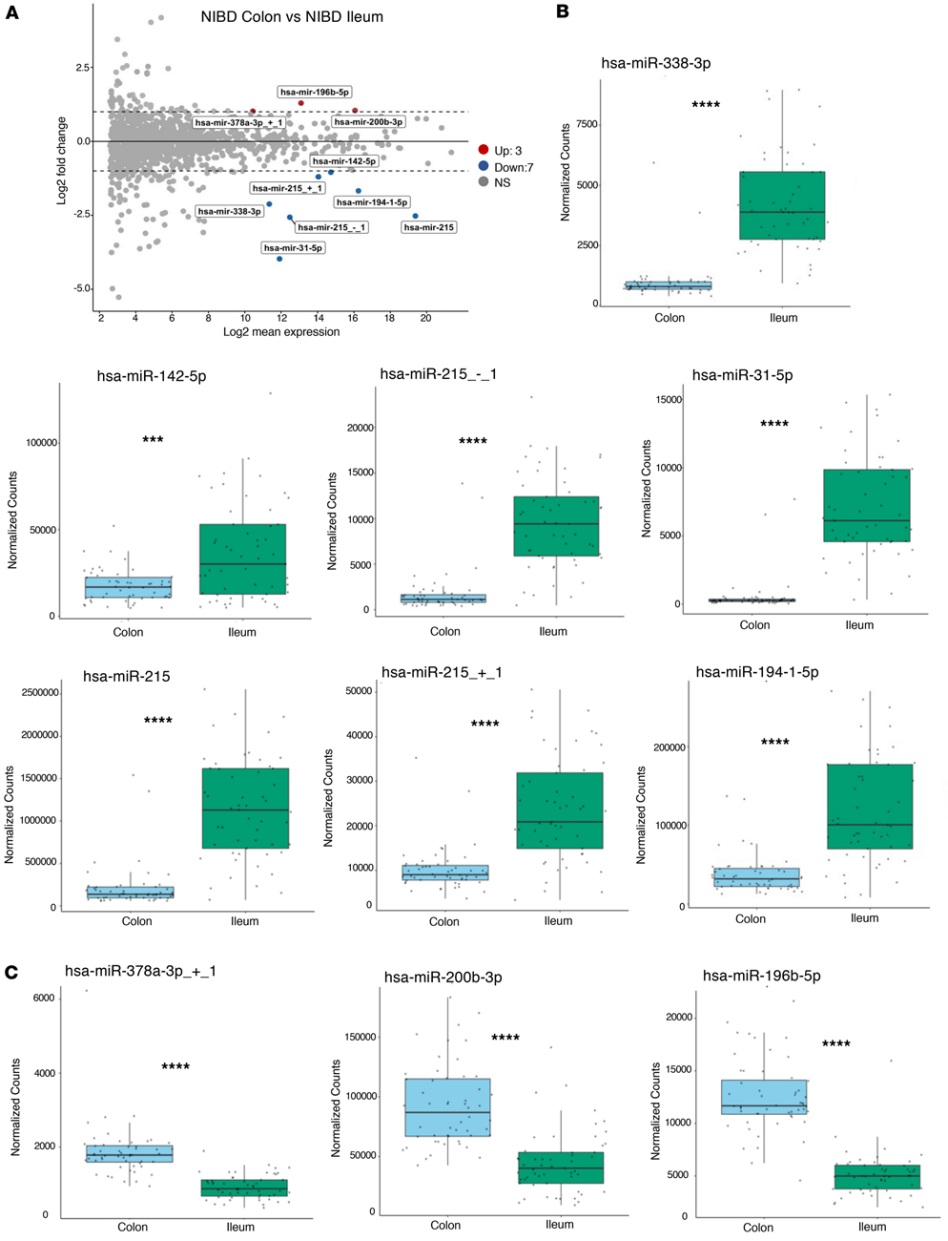
MicroRNA profiles in the colon of pediatric CD separate into 2 clusters. (**A**) MA plot showing miRNAs that are significantly differentially expressed between patients with colonic and ileal NIBD (*n* = 105). Dashed lines represent the log_2_ fold change (log_2_FC) of expression –1.0/+1.0 (horizontal). Up- or downregulated miRNAs are colored red or blue, respectively, with an adjusted *P* < 0.05 and baseMean > 1,000. (**B** and **C**) Box-and-whisker plots of the normalized read counts of 7 miRNAs significantly enriched in the ileum (**B**) and 3 miRNAs significantly enriched in the colon (**C**). Whiskers mark minimum and maximum, the borders of the box mark the upper and lower 25th quartile, and the horizontal line in the box indicates the median. Each data point represents a patient sample. ***P < 0.001, ****P < 0.0001; Student’s *t* test and Mann-Whitney *U* test.

**Figure 3:**
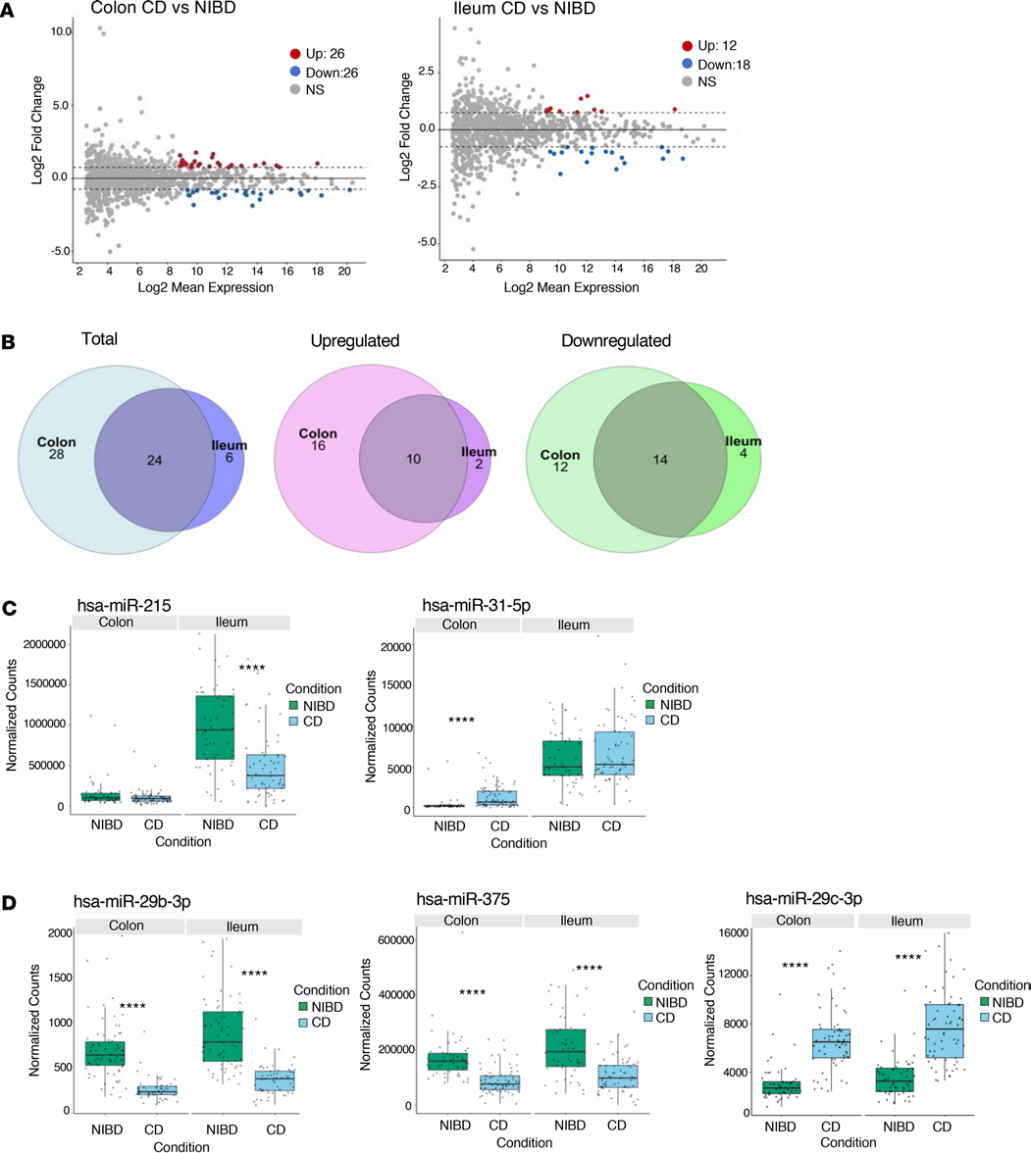
Many microRNAs significantly altered in pediatric CD are shared between the 2 tissue types. (**A**) MA plot showing miRNAs that are significantly differentially expressed between patients with CD and NIBD (*n* = 245) in the colon (left) and ileum (right). Dashed lines represent the log_2_ fold-change of expression –0.75/+0.75 (horizontal). Up- or downregulated miRNAs are colored red or blue, respectively, with an adjusted *P* < 0.05 and baseMean > 500. (**B**) Venn diagrams of significantly altered pediatric miRNAs (baseMean > 500, adjusted *P* < 0.05, log_2_FC > 0.75 or < –0.75) in ileum and in colon: total (left), downregulated (middle), and upregulated (right). Paralogs are listed as one miRNA. (**C**) Box-and-whisker plots of the normalized counts of miR-215 and miR-31, which are 2 of the miRNAs significantly differentially expressed specific to the colon or ileal tissue samples. (**D**) A comparison of normalized counts for miR-29b, miR-375, and miR-29c, which are found to be significantly altered in both ileum and colon tissue from pediatric patients with CD. Whiskers mark minimum and maximum, the borders of the box mark the upper and lower 25th quartile, and the horizontal line in the box indicates the median. Each data point represents a patient sample. ****P < 0.0001; Wald test and Mann-Whitney *U* test.

**Figure 4:**
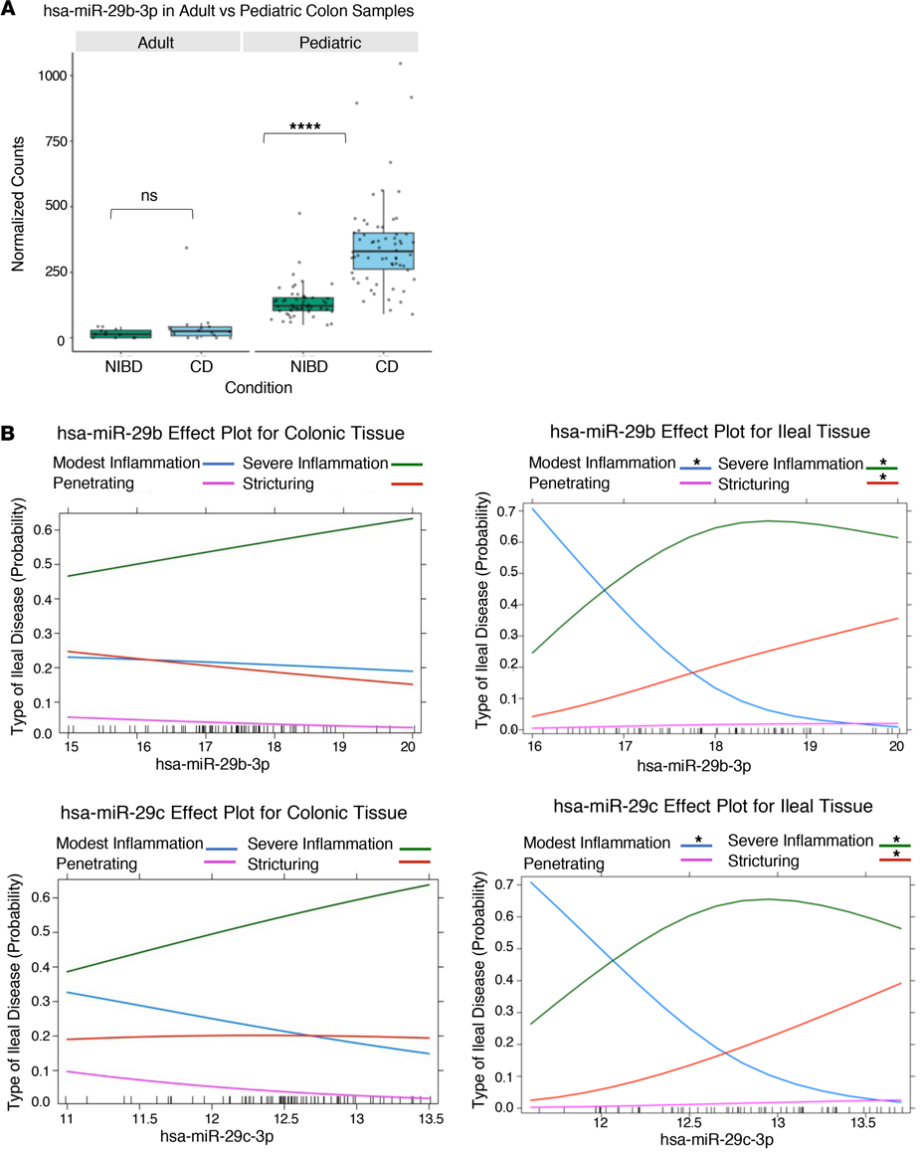
Index levels of ileal miR-29b/c are associated with the development of severe phenotypes in pediatric patients with CD. (**A**) Normalized counts of miR-29b in colon tissue from pediatric and adult patients (*n* = 142). Each data point represents a patient sample. (**B**) Effects plots for miR-29b (top) and miR-29c (bottom) with the VST-transformed counts for each microRNA on the *x* axis and the probability of association with type of ileal disease on the *y* axis (*n* = 75 for colon, *n* = 65 for ileum). Multinomial logistic regression was performed with VST-transformed counts for each miRNA and type of ileal disease for each patient (modest inflammation used as a reference). FDR was used for multiple testing correction. Type of ileal disease is indicated by colors defined within the figure. Adjusted *P* values for each association are placed above the appropriate type of ileal disease. *P < 0.05, ****P < 0.0001; Wald test and Mann-Whitney *U* test.

**Figure 5:**
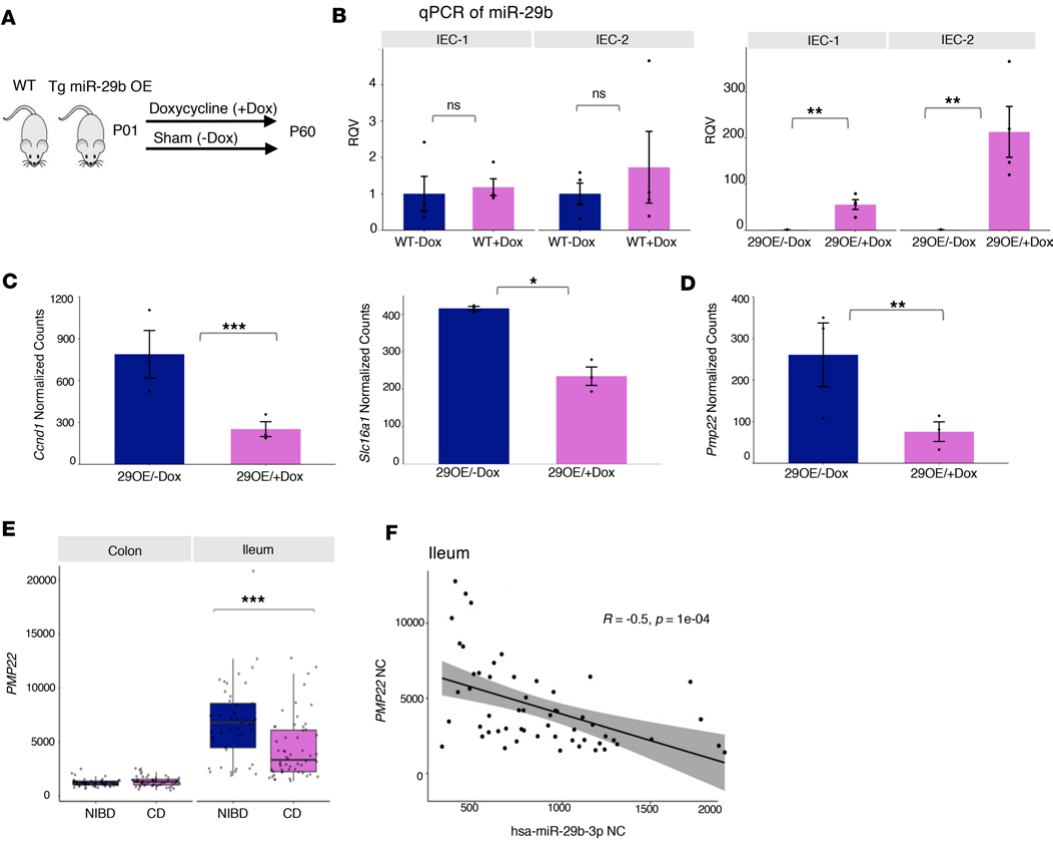
Upregulation of miR-29b is associated with loss of PMP22 in both mouse and human. (**A**) Schematic of Dox-inducible miR-29b overexpressing (29OE) mouse model. (**B**) qPCR showing the relative quantitative value (RQV) of miR-29b in 29OE mice without Dox treatment (29OE/–Dox, *n* = 4) (left) and with Dox treatment (29OE/+Dox, *n* = 4) for IEC-1 and IEC-2 (right). IEC-1 and IEC-2 represent 2 independent intestinal epithelial fractions from the same mice. Dark blue represents the –Dox treatment, and pink represents the +Dox treatment. (**C**) Normalized counts of previously validated miR-29 target genes Ccnd1 and Slc16a1 in the transgenic mouse model. Dark blue represents the –Dox treatment (*n* = 3), and pink represents the +Dox treatment (*n* = 3). (**D**) Normalized counts of miR-29 predicted target gene, Pmp22, in the transgenic mouse model. Dark blue represents the –Dox treatment (*n* = 3), and pink represents the +Dox treatment (*n* = 3). (**E**) Normalized counts of miR-29 predicted target gene PMP22 in pediatric CD and NIBD in both tissue types. (**F**) Correlation of miR-29b (*x* axis) and PMP22 (*y* axis) in pediatric ileum samples. Data presented as mean ± SEM. *P < 0.05, **P < 0.01, ***P < 0.001; Student’s *t* test and Mann-Whitney *U* test.

**Figure 6:**
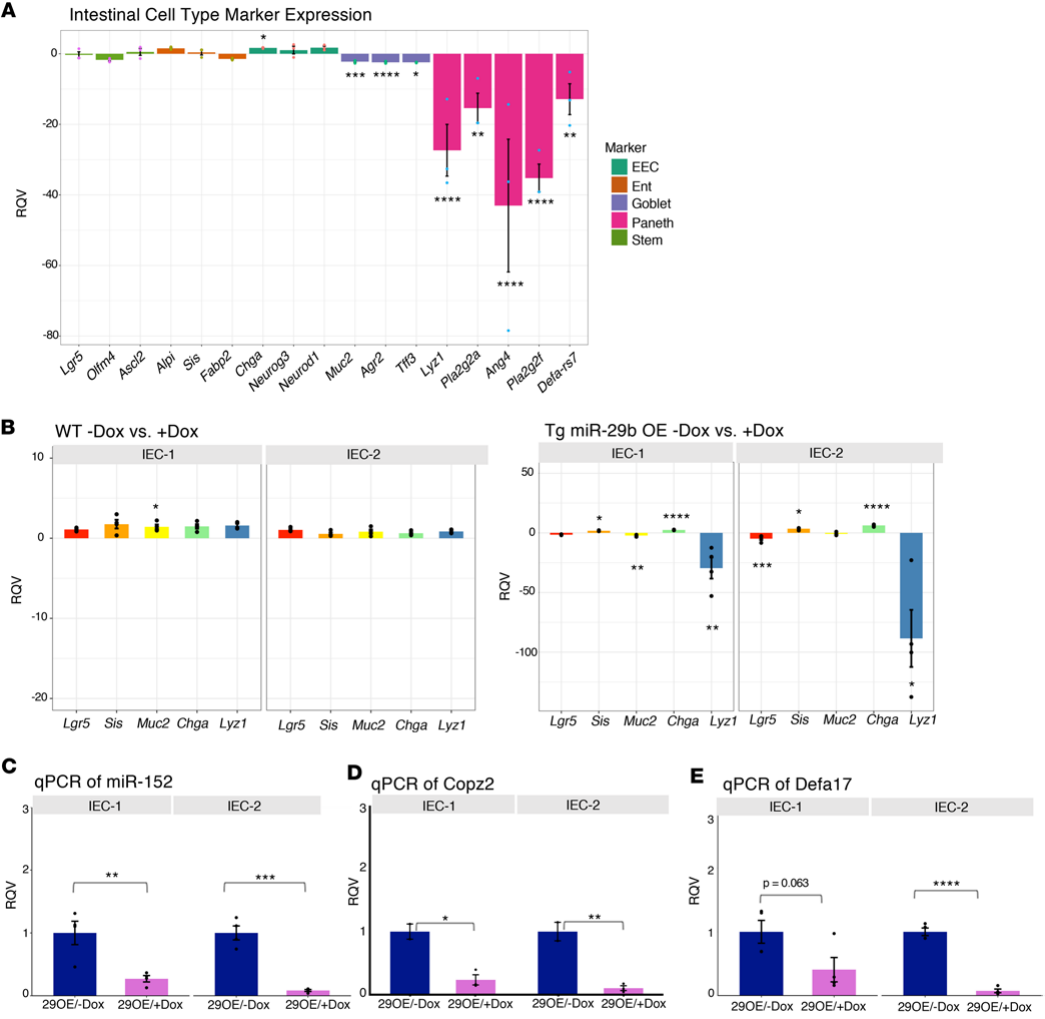
Overexpression of miR-29b in mice leads to dramatic reduction of Paneth cell gene markers. (**A**) RNA-Seq data from the jejunum shows relative quantitative value (RQV) (*y* axis) of intestinal epithelial cell type markers (*x* axis) in 29OE/+Dox (*n* = 3) versus 29OE/–Dox (*n* = 3). The markers for each cell type are colored as shown in the figure. *P < 0.05, **P < 0.01, ***P < 0.001, ****P < 0.0001; Wald Test. (**B**) qPCR of 29OE/–Dox (left, *n* = 4) and 29OE/+Dox (right, *n* = 4) showing the RQV for marker genes of intestinal stem cells (Lgr5) and 4 different major lineages of the intestinal epithelium (enterocyte, goblet cell, enteroendocrine cell, and Paneth cell) with and without Dox treatment for IEC-1 and IEC-2. (**C**–**E**) qPCR showing the relative quantitative value (RQV) of miR-152 (**C**), Copz2 (**D**), and Defa17 (**E**) 29OE for IEC-1 (*n* = 4,4) and IEC-2 (*n* = 4,4). Dark blue represents the –Dox treatment, and pink represents the +Dox treatment. Data presented as mean ± SEM. *P < 0.05, **P < 0.01, ***P < 0.001, ****P < 0.0001; Student’s *t* test and Mann-Whitney *U* test.

**Figure 7:**
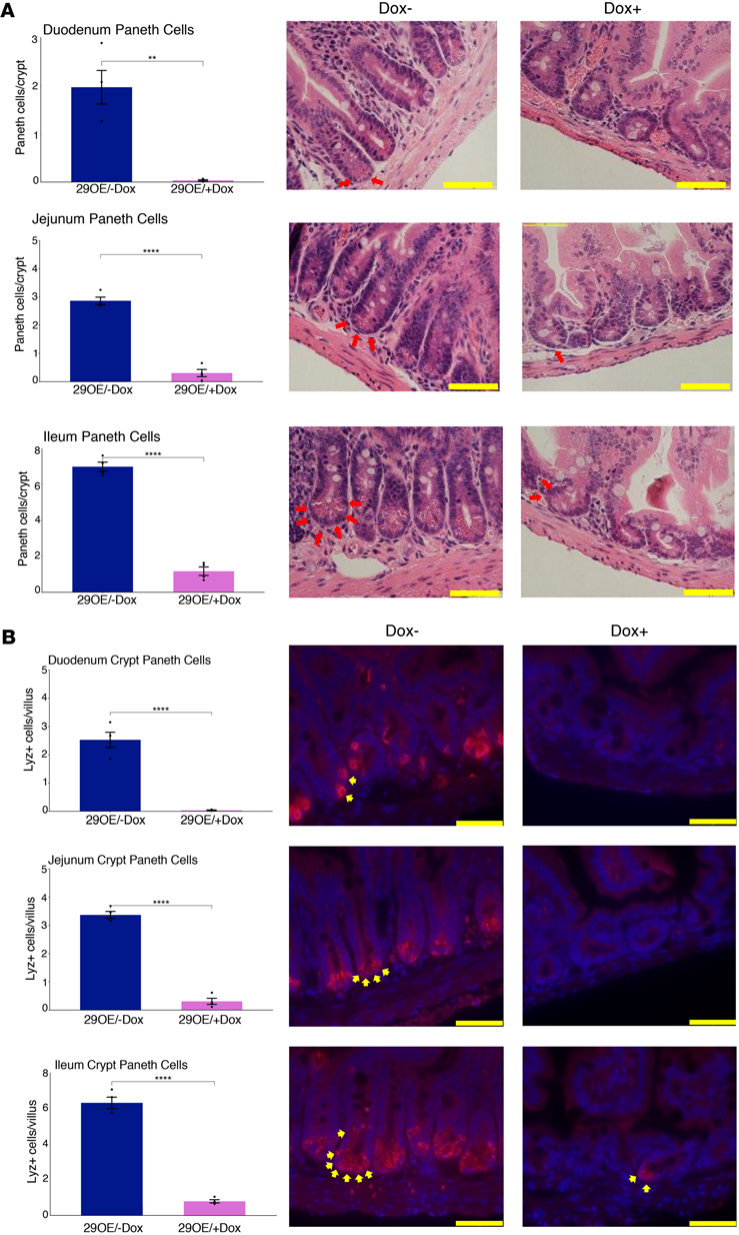
Gain of miR-29b leads to loss of Paneth cells. (**A**) Paneth cell counts per crypt from bright-field H&E-stained tissue sections of 29OE/–Dox (*n* = 4) and 29OE/+Dox (*n* = 4) in the proximal duodenum, midjejunum, and distal ileum (×600 images). (**B**) Paneth cell counts per crypt from Lyz1 immunofluorescent (red) and DAPI fluorescent (blue) tissue sections of 29OE/–Dox (*n* = 4) and 29OE/+Dox (*n* = 4) in the proximal duodenum, midjejunum, and distal ileum (×600 images). Scale bars: 50 μm at ×600 and 100 μm at ×200. Individual Paneth and goblet cells are indicated by red arrows. Dark blue represents the –Dox treatment, and pink represents the +Dox treatment. Data presented as mean ± SEM. **P < 0.01, ****P < 0.0001; Student’s *t* test and Mann-Whitney *U* test.

Our data afford the unique opportunity to define the miRNAs that are enriched in either the human colon or the ileum at baseline, using matched tissue from the same control (NIBD) individuals. Using DESeq2 we found 10 miRNAs significantly differentially abundant between the ileum and colon (Figure 2A) — 7 miRNAs significantly enriched in the ileum (Figure 2B) and 3 miRNAs significantly enriched in the colon (Figure 2C). The ileal-enriched miRNAs included miR-31, which we have previously reported on, most prominently in adult CD (25, 26). The colon-enriched miRNAs include miR-196b, which has long been associated with colonic CD (23, 34) and ulcerative colitis (35, 36). Overall, the results of this analysis show that the miRNA profiles of the ileum and colon from matched patients with NIBD are remarkably similar, with only a small set of discriminative miRNAs.

Next, to identify differentially expressed miRNAs in pediatric CD versus NIBD, we performed analysis with DESeq2 in both tissue types separately. This analysis revealed 30 significantly altered miRNAs in ileum (12 upregulated, 18 downregulated) and 52 in colon (26 upregulated, 26 downregulated) from pediatric patients with CD relative to the corresponding NIBD samples (Figure 3A). Of these, ~40% are overlapping between the 2 tissue types (Figure 3B). Although many of the most robustly differentially expressed miRNAs in pediatric CD are shared between ileum and colon, several notable miRNAs are unique to one or the other tissue type. For example, miR-215 and miR-31 are significantly altered in pediatric CD only in ileum and colon, respectively (Figure 3C). Both of these miRNAs are significantly altered in the colon of adult patients with CD based on our previously published data sets (23, 25). Examples of miRNAs found to be significantly altered in both ileum and colon tissue from pediatric patients with CD are miR-29b, miR-29c, and miR-375 (Figure 3D).

### Index levels of ileal miR-29b/c are associated with the development of severe inflammation and stricturing in pediatric patients with CD.

We sought to determine whether any of the significantly differentially expressed miRNAs in colon or ileum (Figure 3A) are associated with clinical characteristics or are predictive of future disease outcomes (Supplemental Table 1). We first performed binomial regression analysis for all binary outcomes. We found that 8 colonic miRNAs are modestly associated with family history, 1 ileal miRNA isoform (miR-215_-_1) is moderately predictive of surgery with anastomosis, and 7 colonic miRNAs (including the miR-21 family and miR-31) are strongly predictive of rectal or sigmoid involvement (Supplemental Table 2). Both miR-31 and miR-21 have been implicated previously in adult CD as well as in mouse models of colitis. Next, we performed multinomial regression analysis for more complex clinical outcomes and showed that index levels of 8 ileal miRNAs (Table 3) are significantly associated with the development of at least 1 of the ileal disease subtypes: severe inflammation, stricturing, or penetrating. Three of these 8 are significantly associated with both severe inflammation and stricturing (Table 3). Of these 3, only 2 (miR-29b and miR-29c) are not also altered in adult CD, based on our previously published analysis (Figure 4A) (23, 25), suggesting that they are prominent and distinguishing features of pediatric CD. We then performed logistic regression analysis to determine whether index levels of miR-29b and miR-29c are associated with the type of ileal disease that a pediatric patient will develop over time (Figure 4B). We found that increasing index levels of ileal (but not colonic) miR-29b and miR-29c are strongly predictive of severe inflammation and stricturing (Figure 4B).

### Upregulation of miR-29b is associated with loss of gene encoding tight junction protein PMP22 in mice and humans.

To determine the effects of miR-29 upregulation on the intestine, we leveraged a doxycycline-inducible (Dox-inducible) miR-29b overexpressing (29OE) mouse model (Figure 5A). For this study, we believe a whole-body 29OE model is necessary as a starting point because the specific cell types or even tissue layers in the intestine in which miR-29 upregulation occurs in pediatric CD is not known. We first performed histological analysis of duodenal, jejunal, and ileal tissue isolated after 60 days of postnatal Dox administration (29OE/+Dox) and compared with control mice (29OE/–Dox). In 2 separate rounds of analysis (each with *n* = 4 29OE/+Dox and *n* = 4 control mice), we did not observe any gross disturbances in small intestinal architecture nor any substantial differences in intestinal crypt depth or density in 29OE/+Dox relative to control (Supplemental Figure 3).

Since a compromised epithelial barrier is one of the hallmark features of CD, we next isolated intestinal epithelial cells (IECs), and we confirmed that miR-29b levels are significantly elevated in 29OE/+Dox mice relative to control (Figure 5B) and that this induction is dependent upon the miR-29 overexpression cassette; it is not due to Dox treatment alone (Figure 5B). RNA-Seq analysis of IECs in 29OE/+Dox relative to control showed significant downregulation of 72 genes, including very well-established miR-29 target genes such as Ccnd1 and Slc16a1 (Figure 5C) (37, 38). These data confirm, at the gene expression level, the expected gain-of-function of miR-29 in the intestinal epithelium in the 29OE/+Dox mice.

Notably, we also found that Pmp22, a predicted miR-29 target gene, is among the most significantly downregulated genes in 29OE/+Dox mice (Figure 5D). This gene encodes a tight junction protein that very recently was shown to promote intestinal barrier function (39). Although miR-29 has been implicated in the control of barrier capacity through regulation of the tight junction protein Cldn1, Pmp22 has not been reported as a miR-29 target in the intestine (40, 41).

To determine whether this regulatory relationship holds in humans, we analyzed our previously reported RNA-Seq data from a majority subset of the same human samples used in this study (*n* = 203) (Supplemental Figure 4) (unpublished observations). We found that PMP22 is among only 16 genes that are significantly downregulated in both the ileum of pediatric patients with CD (relative to NIBD controls) and in IECs of 29OE/+Dox mice (relative to 29OE/–Dox controls) (Supplemental Table 3). Moreover, we observed that PMP22 is much more highly expressed in pediatric human ileum compared with colon (Figure 5E) and also is significantly suppressed only in the ileum and not the colon of pediatric patients with CD compared with NIBD controls (Figure 5E). Notably, we also observed that miR-29b levels are highly significantly inversely correlated with PMP22 in the ileum of pediatric patients with CD (Figure 5F). Taken together, these data point to a miR-29 target in the small intestine, the reduction of which — in the context of miR-29 upregulation — may contribute to the compromised barrier observed in pediatric CD.

### Overexpression of miR-29b in mice leads to dramatic reduction of Paneth cell gene markers.

Upon further analysis of the murine RNA-Seq data from the jejunum, we observed dramatic downregulation of 5 major Paneth cell markers (Figure 6A). Contrastingly, we observed only modest effects on goblet and enteroendocrine cell markers (Figure 6A) and observed very little influence on stem or enterocyte markers (Figure 6A). We then measured by quantitative PCR (qPCR) the levels of marker genes of intestinal stem cells (Lgr5) and 4 different major lineages of the intestinal epithelium (enterocyte, goblet, enteroendocrine, and Paneth). The most dramatic effect was observed for a classic marker of crypt-based Paneth cells, lysozyme 1 (Lyz1), downregulated by more than 20-fold in 29OE/+Dox relative to control (Figure 6B). No significant change in Lyz1 or any other marker was detected in WT mice (without the miR-29b overexpression cassette) treated with Dox for the same duration of time (Figure 6B). qPCR for additional Paneth cell markers Defa17, miR-152, and Copz2 in IECs revealed a similar downregulation in 29OE/+Dox relative to control (Figure 6, C–E). The latter 2 are particularly informative, since they are not thought to be physically associated with granules, unlike Defa17 and Lyz1, suggesting that there is a loss of Paneth cells and not merely a granulation defect.

### Gain of miR-29b leads to loss of Paneth cells in mice.

Matching the results from the transcriptomic study, H&E analysis showed that the number of granulated cells (which we use as a proxy for Paneth cells) per crypt is significantly reduced in 29OE/+Dox mice compared with 29OE/–Dox controls (Figure 7A). We next performed Lyz1 immunofluorescence (IF) analysis, which showed an even more dramatic loss of canonical Paneth cells (Figure 7B). H&E and IF analyses in an independent cohort of mice confirmed these results (Supplemental Figure 5). Alcian blue staining revealed only a comparatively modest effect of miR-29b overexpression on goblet cell number in both crypts (Supplemental Figure 6A) and villi (Supplemental Figure 6B), consistent with the results of the gene marker analysis (Figure 6A). These findings were not observed in the H&E analysis for WT mice (without the miR-29b overexpression cassette) treated with Dox for the same duration of time (Supplemental Figure 7).

### miR-29b/c levels are linked to Paneth cell number in pediatric patients with CD.

**Figure 8:**
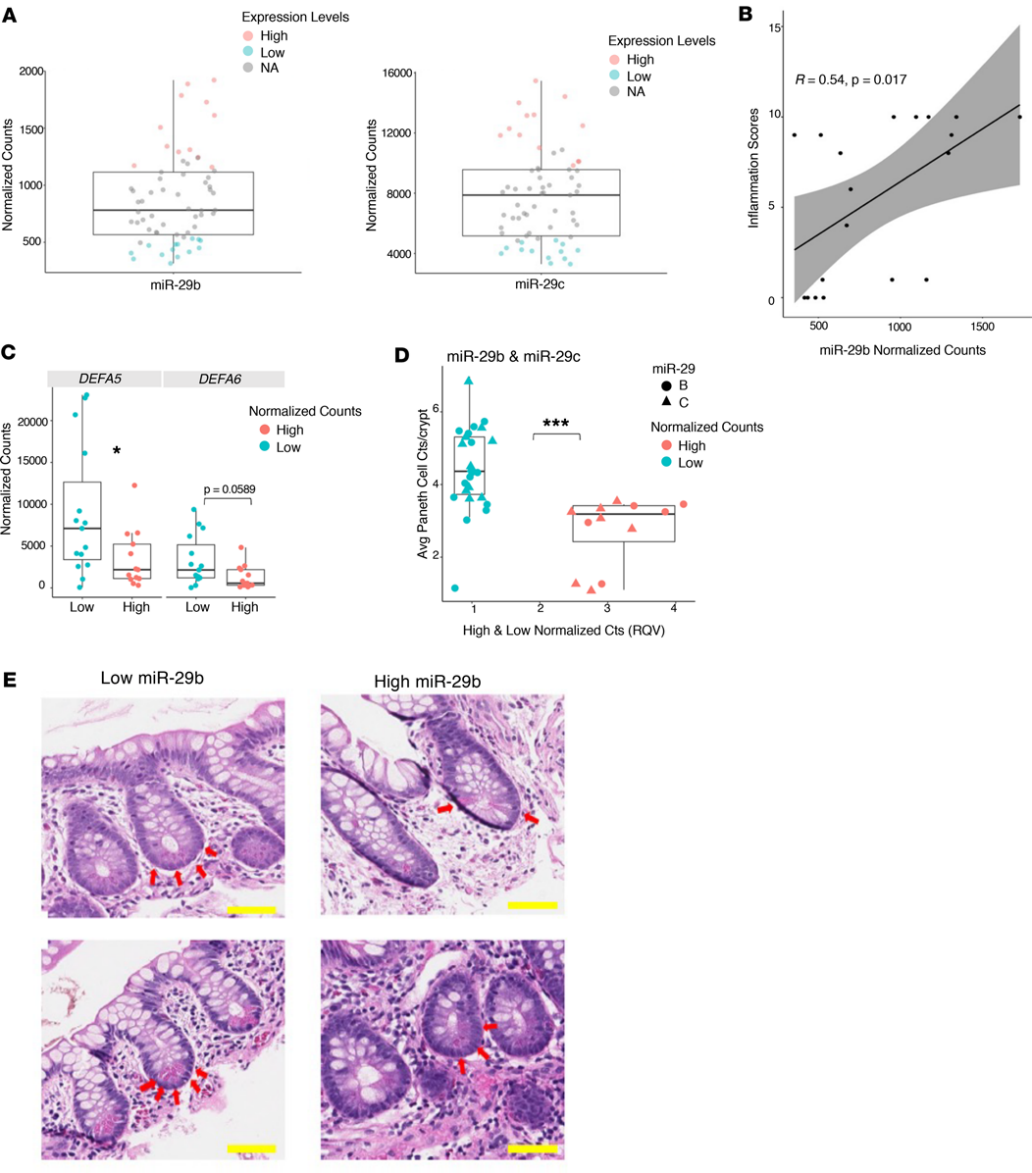
High levels of miR-29b/c are linked to low numbers of Paneth cells in pediatric patients with CD. (**A**) The normalized counts of miR-29b/c in the selected High-29 (*n* = 20) or Low-29 (*n* = 19) groups. Each data point represents a patient sample. (**B**) Correlation of miR-29b (*y* axis) and inflammation scores (*x* axis) across patients in the pediatric High-29 and Low-29 groups (High-29, *n* = 10; Low-29, *n* = 9). (**C**) Normalized counts of miR-29b/c in High-29 and Low-29 samples for both DEFA5 and DEFA6. (**D**) Relative quantitative values (RQV) of miR-29b/c expression in High-29 and Low-29 samples (*x* axis) and average Paneth cell counts per crypt (*y* axis). (**E**) Two representative bright-field H&E-stained images of ileal crypts from High-29 and Low-29 patients (×600). Scale bar: 50 μm. Each data point represents a patient sample. *P < 0.05; ***P < 0.001; Student’s *t* test and Mann-Whitney *U* test.

Based on the functional studies in mice, we hypothesized that miR-29b/c levels are correlated with Paneth cell number in pediatric patients with CD. To test this hypothesis, we first selected the patients with the highest or lowest levels of miR-29b/c, termed High-29 (*n* = 20) or Low-29 (*n* = 19), respectively (Figure 8A). Among these, 9 samples dropped out of further analysis due to an inability to meet our histology criterion for displaying at least 10 well-oriented crypts with fully discernible crypt bottoms. Of the remaining 30 samples, 19 (High-29, *n* = 10; Low-29, *n* = 9) were analyzed for inflammation. We found that miR-29b levels are significantly correlated with inflammation score (Figure 8B). High-29 samples were found to have significantly reduced levels of DEFA5 and DEFA6, which are specific markers of Paneth cells (Figure 8C and Table 4). The samples (*n* = 30) were then subject to H&E analysis, which revealed that the High-29 group is associated with significantly fewer Paneth cells per crypt (Figure 8, D and E). Taken together with the previous results, these findings are strongly indicative of a dominant regulatory effect of miR-29b on Paneth cells in mice and humans.

## Discussion

This study represents a comprehensive miRNA analysis of a large cohort of pediatric patients with CD. Major strengths of this study include: (a) large sample size of treatment-naive patients (*n* = 245); (b) patient-matched ileal and colonic tissue; (c) detailed regression analysis with clinical characteristics; (d) discovery of index biopsy miRNA indicators of disease outcomes; and (e) mouse and human studies linking aberrant miR-29 with the loss of Paneth cells and altered PMP22 expression. A primary finding is that miR-29 and possibly other miRNAs could be used as prognostic indicators of disease subtype and/or severity. Major open questions that our study does not address include the following. (a) How is miR-29 misregulated in pediatric CD? (b) Are the effects of miR-29 overexpression on Paneth cells developmental phenotypes? (c) In what intestinal cell types or mucosal layers is miR-29 overexpression the most dominant and functionally relevant in the context of pediatric CD? These questions warrant further investigation in follow-up studies.

The answers to the third question listed above will be particularly valuable for determining the downstream targets of miR-29 that are most critical in the context of pediatric CD. In the murine RNA-Seq data presented in this study, none of the genes encoding canonical regulators of Paneth cell differentiation (42) (Sox9, Atoh1, Erbb3), appear to be affected in the intestinal epithelium upon miR-29 overexpression. Intriguingly, though, we do observe a dramatic decrease in Ciita, which codes for the master transcriptional regulator of MHC Class II genes (43). Ciita is a predicted target of miR-29, and loss of MHC Class II activity specifically in intestinal stem cells can lead to significantly reduced secretory cell allocation (44). Taken together, these data raise the possibility that upregulation of miR-29 leads to increased direct targeting and suppression of Ciita, which in turn reduces MHC Class II signaling and Paneth cell differentiation. This hypothesis requires extensive rigorous evaluation that is well outside the scope of the present study, but we believe it merits future investigation. It is also quite possible that the upregulation of miR-29 in pediatric CD is greatest in cells from layers beneath the epithelium, including fibroblasts, lymphatic endothelial cells, telocytes, immune cells, and/or enteric neurons. For example, a possibility is that increased miR-29 specifically in lamina propria T cells leads to reduced DNMT3A, a well-established miR-29 direct target, which would decrease promoter methylation of the IFN-γ gene (IFNG), increase IFN-γ levels, and promote chronic inflammation (45, 46). We propose that future studies should focus on determining the cell types driving the aberrant miR-29 signal in pediatric CD in order to uncover direct targets and molecular mechanisms that underpin the association of miR-29 with inflammation, stricturing, and/or Paneth cell loss.

miR-29 has received attention previously as a potent regulator of several gastrointestinal phenotypes (15, 47). A few different studies of IBS have shown that miR-29 promotes gut permeability in mice by directly targeting and suppressing tight junction proteins (40, 48, 49). In this context, upregulation of miR-29 would severely compromise the gut barrier and thereby promote IBS disease severity. In other work, though, it has been demonstrated that miR-29 suppresses intestinal fibrosis and, therefore, could be a protective factor in IBD (50, 51). Moreover, another study has shown that loss of miR-29 may exacerbate inflammatory phenotypes in the intestine (52). In fact, at least 1 study has suggested that a miR-29 mimic is a potential therapeutic for IBD, especially in cases of severe inflammation (53). These separate reports of antiinflammatory, antifibrotic, and yet barrier-compromising functions paint a highly multifaceted picture of miR-29 in the gut. As it pertains to IBD, it is possible that miR-29 has both antagonistic and protective functions, depending on the cellular context, disease etiology, age of onset, and/or time point during disease progression.

Our discovery that miR-29 may also suppress Paneth cells provides another means by which gut permeability and inflammation may increase. Specifically, we suggest that an early increase in miR-29 leads to Paneth cell loss, which dampens antimicrobial activity, likely promotes small intestinal dysbiosis, in turn compromising barrier integrity and leading to increased risk of inflammation. Paneth cell defects have long been implicated in the pathogenesis of CD and, in fact, are more prevalent in children compared with adults (54, 55). We suggest that the aberrant elevation of miR-29 in pediatric CD, but not adult CD, may contribute to this difference. Our findings add to the rich and complex web of intestinal miR-29 behavior.

Several reports have called for the investigation of both miR-29 mimics and inhibitors as potential therapeutics in IBD and related chronic disorders of the gut. However, we strongly urge caution, as miR-29–based therapeutics are likely to be challenging, given the context-specific functions of miR-29 described above. Further work is needed to sort out the regulatory effects of miR-29 in distinct cell types of the intestine during different stages of disease progression. We are even more intrigued by the potential of miR-29–based therapeutics for acute gut conditions (such as microbial infections), or conditions such as necrotizing enterocolitis, for which Paneth cell defects are critical to pathogenesis. For example, is miR-29 strongly upregulated after *Salmonella* or *Listeria* infection, and if so, could that be responsible, at least in part, for the Paneth cell defects reported under those conditions? We believe that such questions, while outside scope of the present study, merit detailed investigation in the future.

The mechanistic underpinnings of pediatric CD are still poorly understood. We believe this study marks an important turning point in the investigation of miRNAs in pediatric CD and provides a rich resource to the research community for the identification of key regulators of the disease, well beyond miR-29. For example, our data point to a significant loss of miR-375 in CD. At least 1 prior study showed that the loss of miR-375 upregulates proinflammatory factors such as TLR4 and NF-κB (56). We believe that more detailed investigation of the mechanisms by which miR-375 and other miRNAs revealed by this data set might control the inflammatory, stricturing, or other IBD phenotypes is warranted. Such studies may uncover novel and effective therapeutic avenues for pediatric and/or adult CD.

## Methods

### Sex as a biological variable.

Our study examined female and male pediatric patients and accounted for this covariate in our analyses. We did not observe sex-specific differences in miR-29 expression or other findings. Therefore, we restricted the mouse study to 1 sex (male) for tractability and cost purposes. It is unknown whether the findings are relevant for female mice.

### Patient population and sample acquisition.

Patient samples were acquired from the University of North Carolina Multidisciplinary IBD Center. All samples were procured from treatment-naive pediatric patients undergoing endoscopy for suspicion of IBD. NIBD control samples were defined as patients with biopsies that were subsequently identified as histologically normal. Clinical information gathered in this study include demographic and clinical variables: family history, age, sex, disease duration, and disease location. Information from patient clinical parameters is provided in Supplemental Table 1. From formalin-fixed paraffin-embedded (FFPE) tissue, mucosal biopsies were obtained during endoscopy from macroscopically unaffected sections of the ascending colon and terminal ileum at the time of surgery. These index biopsies were also confirmed by a pathologist to have no active inflammation. Microscopic inflammation was defined as (a) neutrophilic infiltration of crypt epithelium and crypt abscess formation and/or (b) architectural distortion and basal lymphoplasmacytosis of the lamina propria. Stricturing and fistulizing phenotypes were defined using endoscopy and/or imaging (fluoroscopy, CT, or MR) and correlated with patient symptomatology, in comparison with a nonstricturing, nonfistulizing phenotype. Stricturing and fistulizing phenotypes were recorded at time of follow-up, with a mean follow-up of 6 years.

### smRNA library preparation and sequencing.

RNA was isolated from FFPE tissue using the Roche High Pure miRNA Isolation Kit following the manufacturer’s protocol as previously described (25). RNA purity was quantified with the NanoDrop 2000 instrument (Thermo Fisher Scientific), and RNA integrity was quantified with the Agilent 2100 Bioanalyzer (Aglient Technologies). smRNA libraries were generated using the TruSeq Small RNA Sample Preparation Kit (Illumina). Sequencing (Single-end, 50 bp) was performed on the HiSeq 2500 platform (Illumina) at the Genome Sequencing Facility of the Greehey Children’s Cancer Research Institute (University of Texas Health Science Center, San Antonio, Texas, USA). Previously published raw sequencing data can be accessed through Gene Expression Omnibus (GEO) accession no. GSE101819. Additional samples are located through GEO accession no. GSE221261.

### smRNA-Seq analysis.

Read quality was assessed using FastQC. miRquant 2.0 (57) was used for read trimming, miRNA annotation, and quantification. Briefly, reads were trimmed using Cutadapt and were then aligned to the human genome (hg19) using both 2 different mapping tools (Bowtie and SHRiMP; refs. 58, 59), and raw miRNA counts were quantified and normalized using DESeq2 (60) to determine significance. Samples with fewer than 1 million reads mapping to miRNAs were removed from further analysis.

Both grouped age (very early onset [VEO] ≤ 6 years; child = 7–12 years; teen = 13–17 years), SIM, and sex of the pediatric samples were accounted for using the limma (61) function removeBatchEffect. After correcting for covariates, PCA analysis was performed using the log normalized DESeq2 (60) values and the plotPCA (60) function in R. Hierarchical clustering was performed using the log normalized DESeq2 values. Using the stats function dist, the Euclidean distance between samples was computed based on expression and plotted with pheatmap. Individual PCAs were computed for both colonic and ileal tissue types using log normalized DESeq2 (60) values with SIM, grouped age, and sex accounted for as covariate. MA plots were created after correcting for covariates and using ggmaplot from ggpubr (62) with parameters set for baseMean, log2FC, and padj.

### SIM.

Length distribution values of all trimmed reads were obtained using miRquant2.0. Reads between 18 and 24 nucleotides are enriched for miRNAs, tRNA fragments, and Y-RNA–derived smRNAs. Reads between 30 and 33 nucleotides are enriched for tRNA halves and Y-RNA derived smRNAs. Background signal is defined as the percentage of reads outside of the 18–24 and 30–33 nucleotide size windows. SIM is calculated by dividing the percentage of reads between 18–24 and 30–33 nucleotides by the background signal.

### Regression analysis.

Associations of differentially expressed miRNAs from both colonic and ileal tissue with binary clinical parameters were explored using generalized linear models (GLM). With categorical variables from clinical data, probabilities were obtained using the multinom function from “nnet” (63) that produced fitted values from a multinomial regression model. Multiple testing correction was performed using FDR adjustment.

### Mouse models.

The miR-29 overexpression mouse model used in this study was first described in ref. 64. The mice were provided by the Deshmukh lab at The University of North Carolina.

### Jejunal epithelial cell preparation.

Harvested small intestine from Dox-exposed and unexposed WT and miR-29b overexpressing mice was measured and divided into 3 equal segments. The middle region was considered jejunum. Subsequent to luminal flushing with ice-cold phosphate buffered saline (PBS), the tissue was longitudinally cut and subjected to incubation in 3 mM EDTA in ice-cold PBS with 1% (v/v) primocin (InvivoGen) for 15 minutes at 4°C. The mucosa of the intestinal pieces was gently scraped of mucus, shaken in ice-cold PBS with 1% (v/v) primocin (InvivoGen) for 2 minutes, and incubated in fresh 3 mM EDTA in ice-cold PBS with 1% (v/v) primocin (InvivoGen) for 40 minutes at 4°C. After 2–6 minutes of gentle manual shaking in ice-cold PBS with 1% (v/v) primocin (InvivoGen), the intestinal pieces were inspected microscopically (magnification, ×100) for detached intestinal crypts and villi, and then diluted 1:2 with ice-cold PBS with 1% (v/v) primocin (InvivoGen). Material that filtered through a 70 μm cell strainer was collected and referred to as jejunal epithelial cell fraction 1 (IEC-1), while material that was collected with washing of the cell strainer surface with ice-cold PBS with 1% (v/v) primocin (InvivoGen) was referred to as IEC-2. IEC-1 and IEC-2 preparations were then pelleted by centrifugation at 110*g* for 10 minutes at 4°C. For RNA extraction, collected pellets were resuspended in 200 μL of lysis buffer (Buffer RL, Norgen Biotek), vortexed for 10 seconds, and stored at –80°C.

### RNA extraction and qPCR.

Total RNA was isolated using the Total RNA Purification kit (Norgen Biotek). TaqMan miRNA Reverse Transcription kit (Invitrogen) was used for reverse transcription of miRNA. High Capacity RNA to cDNA kit (Invitrogen) was used for reverse transcription of RNA for gene expression analysis. Both miRNA and gene expression qPCR were performed using TaqMan assays (Invitrogen) with either TaqMan Universal PCR Master Mix (miRNA qPCR) or TaqMan Gene Expression Master Mix (mRNA qPCR) per the manufacturer’s protocol on a Bio-Rad CFX96 Touch Real Time PCR Detection System. Reactions were performed in duplicate or triplicate using either U6 (miRNA qPCR) (Invitrogen, assay ID: 001973) or Rps9 (mouse mRNA qPCR) (Invitrogen, assay ID: Mm00850060_s1) as the normalizer. miRNA expression was assayed with use of the following probes: hsa-miR-29b (Invitrogen, assay ID: 000413) and hsa-miR-152 (Invitrogen, assay ID: 000475). Gene expression was assayed with use of the following probes: Chga (Invitrogen, assay ID: Mm00514341_m1), Copz2 (Invitrogen, assay ID: Mm04203911_m1), Defa17 (Invitrogen, assay ID: Mm04205962_gH), Lgr5 (Invitrogen, assay ID: Mm00438890_m1), Lyz1 (Invitrogen, assay ID: Mm00657323_m1), Muc2 (Invitrogen, assay ID: Mm01276696_m1), and Sis (Invitrogen, assay ID: Mm01210305_m1). Any undetectable samples were not included in the analysis.

### Mouse RNA library preparation, sequencing, and analysis.

RNA-Seq libraries were prepared using the total extracted RNA from the IEC-1 preparations of Dox-exposed and unexposed miR-29b-overexpressing mice. RNA was quantified with the NanoDrop 2000 (Thermo Fisher Scientific), and RNA integrity was assessed by the Agilent 4200 Tapestation (Agilent Technologies). Libraries were prepared by the Cornell TREx facility using NEBNext Ultra II Directional Library Prep Kit (New England Biolabs) with ribosomal RNA depletion. Sequencing was performed on the NextSeq500 platform (Illumina) at the Genomics Facility in the Biotechnology Research Center at Cornell University. Raw sequencing data are available through GEO (accession no. GSE221261). Read quality was assessed using FastQC. RNA-Seq data were mapped to the mm6 genome with STAR (65).Transcripts were quantified with Salmon (66) using GENCODE release 25 transcript annotations. Normalization and differential analyses were then performed using DESeq2 (60).

### Human RNA library preparation, sequencing, and analysis.

Total RNA was isolated from FFPE tissue using Quick-RNA FFPE MiniPrep (Zymo Research). Purification was performed using the MagMAX kit in the KingFisher system (Thermo Fisher Scientific). Sequencing libraries were then prepared through the TruSeq Stranded Total RNA with Ribo-Zero (Illumina). The NovaSeq 6000 platform was used for paired-end (50 bp) sequencing (Illumina). Salmon (66) was then used to quantify transcripts. Samples with low transcript numbers (<25,000) and poor transcript integrity numbers (TIN) were eliminated from further analysis (*n* = 2). Samples that failed to cluster with their respective tissue type (ileum or colon) through PCA were also discarded from analysis (*n* = 5).

PCA analysis accounted for the 3 covariates that contributed to the greatest variation among samples (batch, sex, and TIN). RUVSeq (67) identified additional unwanted variation by accounting for the top 1,000 genes with the lowest variance out of the top 5,000 genes with highest variance; these were dictated as the control genes. Through this analysis, it was determined that 1 factor of unwanted variation should be used in the final analysis, due to the observed variation in DEGs identified by DESeq2 (60), relative log expression plots, and correlation between factors of unwanted variation and the outcome.

### Mouse tissue histology and histological analysis.

Mouse proximal duodenal, midjejunal, and distal ileal tissue were fixed in 4% (v/v) neutral-buffered paraformaldehyde, embedded in paraffin, and cut into 5 μm transverse sections for various staining experiments. H&E staining was performed for morphometric analyses (crypt depth, villus height, and crypt density) and Paneth cell count determination. Alcian blue (pH 2.5) and eosin (AB&E) staining was performed for goblet cell count determination. Paneth cell counts were also determined by immunofluorescence staining of lysozyme. Briefly, after deparaffinization and antigen retrieval with citrate buffer (10 mM citric acid, 0.05% [v/v] Tween 20, pH 6.0), sections were blocked with 10% (v/v) normal goat serum in PBS for 1 hour at room temperature, incubated with rabbit anti-LYZ primary antibody (1:1,000, Abcam, clone EPR2994[2], catalog ab108508) in PBS with 0.1% (w/v) BSA overnight at 4°C, followed by goat anti–rabbit Alexa Fluor 594 secondary antibody (1:1,000, Invitrogen, catalog A-11012) incubation in PBS with 0.1% (w/v) BSA for 1 hour at room temperature. DAPI (0.1 mg/mL in PBS, Invitrogen, catalog D1306) was used to visualize nuclei. Images were captured using a BX53 Olympus scope (Olympus). Paraffin embedding, sectioning, and tissue staining with H&E and AB&E were performed by the Animal Health Diagnostic Care Histology Laboratory at Cornell University. Images were analyzed for histomorphometric measurements, and cell counts were analyzed with ImageJ software (NIH). At least 10 intact, well-sectioned crypts and 10 intact, well-sectioned villi were used for acquiring histomorphometric measurements and cell counts.

### Patient tissue histology and histological analyses.

Two pathologists independently and blindly graded the inflammatory activity (×400) of ileal H&E-stained sections using the following criteria: high degree of inflammation, neutrophilic activity on 7–10/10 high-power fields; intermediate degree of inflammation, neutrophilic activity on 3–6/10 high-power fields; low degree of inflammation, neutrophilic activity on 0–2/10 high-power fields. Using bright-field images at ×600 magnification, the crypt base eosinophilic granulated Paneth cells of at least 10 well-oriented crypts with fully discernible crypt bottoms were counted.

### Statistics.

R software version 4.1.0 was used for these data analyses. All smRNA annotation and quantification was conducted through miRquant (57). RNA-Seq and smRNA-Seq data were analyzed for differential expression using DESeq2 (60), with the Wald test used for hypothesis testing when comparing 2 groups and *P* values FDR adjusted for statistical significance. Significance of differential expression in qPCR experiments was assessed using Student’s *t* test (unpaired, 2 tailed) to compare 2 groups of independent samples. If we were unable to assume normality in the data set, a nonparametric test (Mann-Whitney *U*) was used. Multinomial logistic regression analysis also was performed using R software, and *P* values were adjusted using FDR. All statistical tests used are detailed in the figure legends. *P* < 0.05 was considered statistically significant. In figure panels, unless otherwise noted, quantitative data are reported as an average of biological replicates ± SEM for all mouse studies. For human samples, quantitative data are reported as mean ± SD.

### Study approval.

Patient samples were acquired from the University of North Carolina Multidisciplinary IBD Center, abiding by IRB-approved protocols (Study ID, 15-0024). Written informed consent was received from all participants prior to inclusion in the study. All participants are identified by number and not by name or any protected health information. UNC and Cornell IACUC approval was obtained for all mouse experiments.

### Data availability.

Previously published raw sequencing data can be accessed through GEO accession no. GSE101819. Additional samples are located through GEO accession no. GSE221261. Values for all data points in graphs are reported in the Supporting Data Values file.

## Author contributions

AJS and MTS acquired, analyzed, and interpreted data; prepared figures; and drafted and revised the manuscript. EH and MD developed and established the mouse colony. KC acquired and analyzed the pediatric RNA-Seq data. JWV helped design the SIM. SA helped analyze and interpret data. CB, GL, MRC, MS, LCZ, and SB provided help with tissue acquisition, patient phenotyping, and inflammation score analysis. TSF designed the study, analyzed and interpreted the data, revised the manuscript, and obtained funding. SZS designed the study, acquired and interpreted the data, revised the manuscript, obtained funding, and obtained IRB approval. PS designed the study, analyzed and interpreted the data, drafted and revised the manuscript, obtained funding, obtained IACUC approval, and supervised the study. All authors uphold the integrity of the work, have had final approval of the manuscript in its entirety, and are accountable for all aspects of the work.

## Supplementary Material

Supplemental data

Supporting data values

## Figures and Tables

**Table 4:**
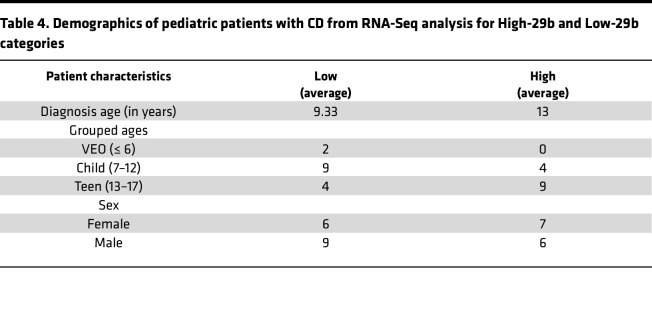
Demographics of pediatric patients with CD from RNA-Seq analysis for High-29b and Low-29b categories

**Table 3:**
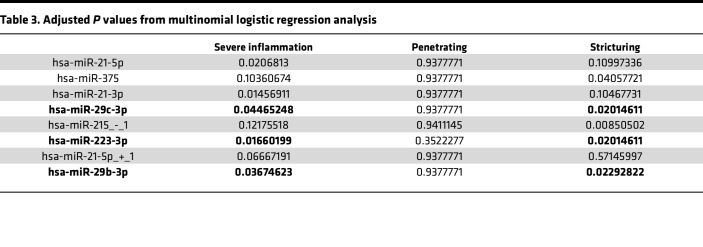
Adjusted *P* values from multinomial logistic regression analysis

**Table 2:**
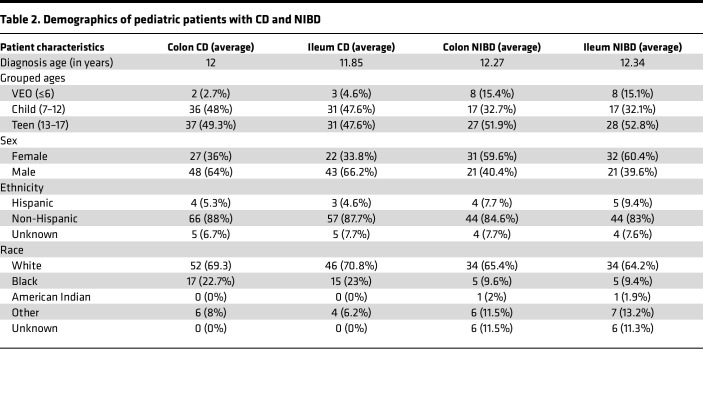
Demographics of pediatric patients with CD and NIBD

**Table 1:**
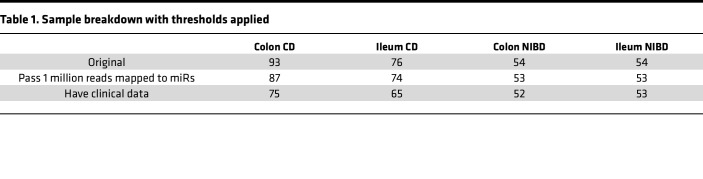
Sample breakdown with thresholds applied

## Version 1 02/22/2024

Electronic publication
