# Supplementary material for: Aberrant miR-29 is a predictive feature of severe phenotypes in pediatric Crohn’s disease
Source: JCI Insight. 2024 Feb 22;9(4):e168800. doi: 10.1172/jci.insight.168800 (PMC10967384; doi:10.1172/jci.insight.168800)
Supplement: Supplemental data [file jciinsight-9-168800-s051.pdf]

Supplemental Figure 1

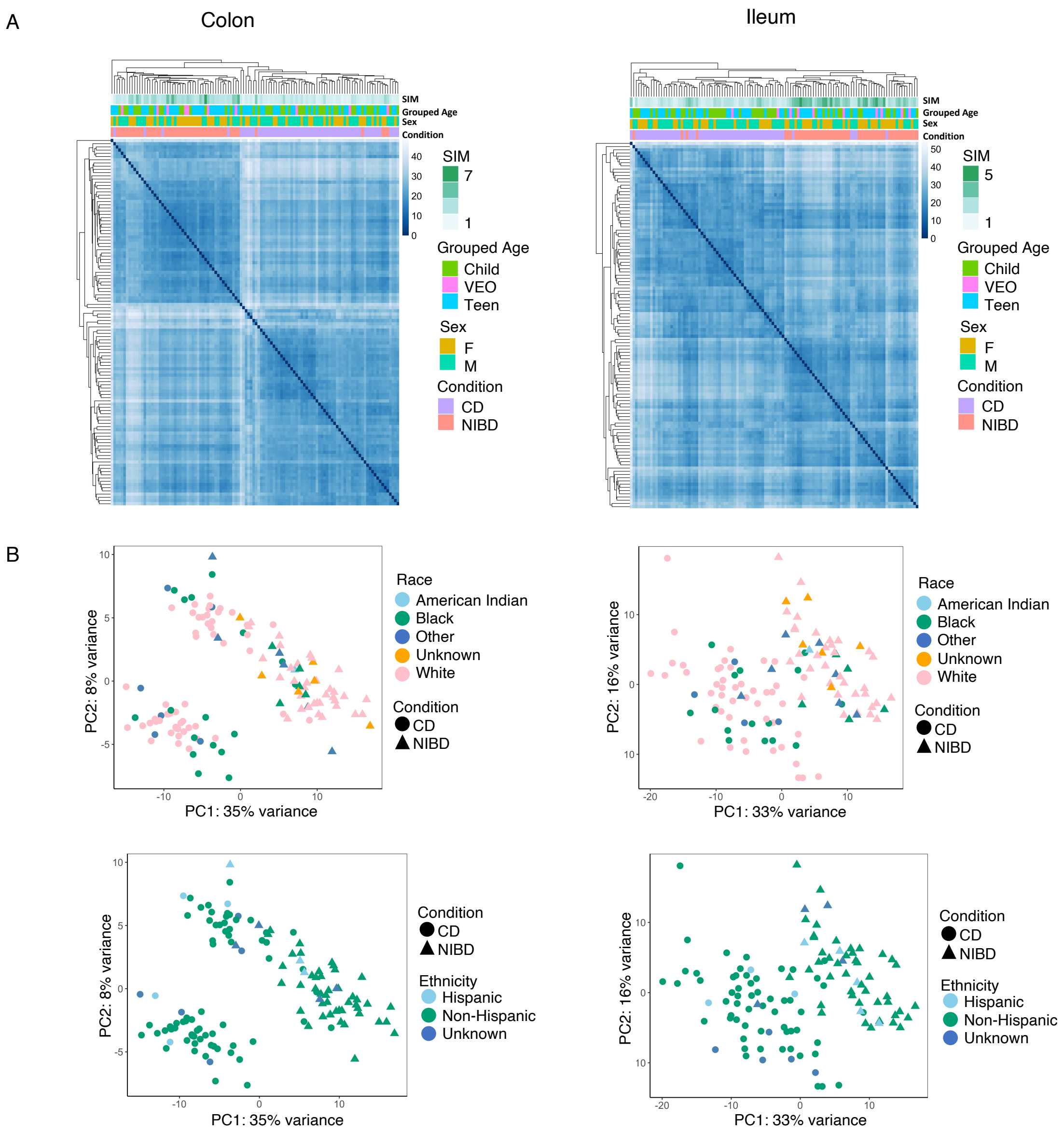

Supplemental Figure 1. (A) Unsupervised hierarchical clustering of the Euclidean distances among patient-matched (n=228) pediatric samples was calculated based on VST normalized counts. The analysis for the colon (left) and ileal tissue (right) account for the covariates of small-RNA integrity metric (SIM), grouped ages, and sex. The CD and NIBD samples are indicated by purple and red boxes. Other covariates are represented as the colors indicated by the legend. (B) PCA plots for colon (left) and ileum (right) in which the patient race and ethnicity information are overlaid in various colors. Disease status is specified by shape.

Supplemental Figure 2

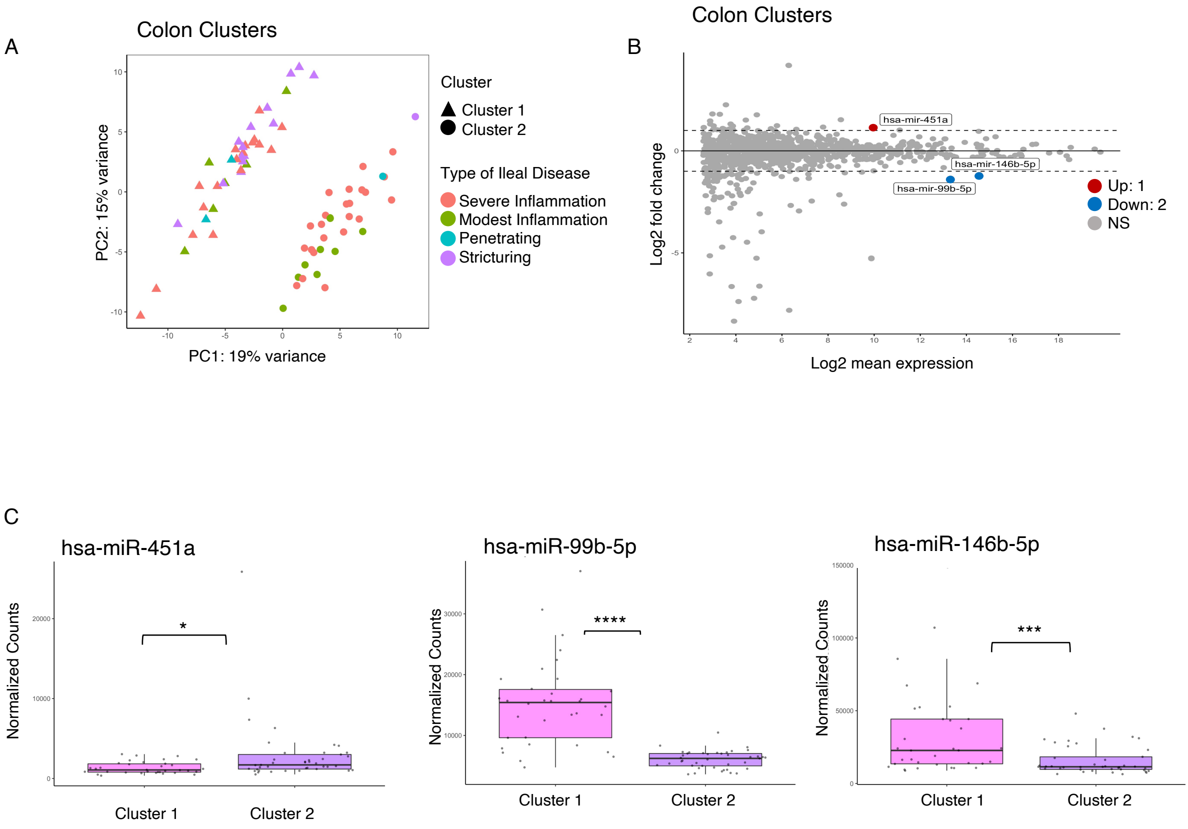

Supplemental Figure 2. Colonic miRNAs separate pediatric CD into two clusters. (A) Principal component analysis (PCA) of VST normalized counts in colon tissue of pediatric CD patients (n=75) accounting for the covariates of small-RNA integrity metric (SIM), grouped ages (VEO < 6, Child = 7-12, Teen = 13-17), and sex. The two colonic clusters are represented by triangles and circles, respectively. Each sample has a color (red, green, blue, purple) according to the type of ileal disease (severe inflammation, modest inflammation, penetrating, stricturing) from each patient. The percent of variation explained is indicated for principal component 1 along the x-axis and principal component 2 along the y-axis. (B) MA plots of differentially expressed miRNAs between colon clusters 1 and 2 (baseMean > 1000, p-adj < 0.05, log2FC > 1 or -1). Dashed lines represent the log2 fold-change of expression –1.0/+1.0 (horizontal). Up- or downregulated miRNAs are colored red (up) or blue (down), with an adjusted p-val < 0.05 and baseMean > 1000. (C) A comparison of normalized read counts of three miRNAs significantly enriched in one of the colon clusters. Box-and-whiskers plots of the normalized read counts of seven miRNAs significantly enriched in the ileum and three miRNAs (C) significantly enriched in the colon. Whiskers mark minimum and maximum, the borders of the box mark the upper and lower 25th quartile, and the horizontal line in the box indicates the median. Each data point represents a patient sample. Pink represents cluster 1 and purple represents cluster 2. (* p < 0.05, *** p< 0.001, **** p< 0.0001; Student's t-test).

Supplemental Figure 3 - 29OE First Round Histomorphometry

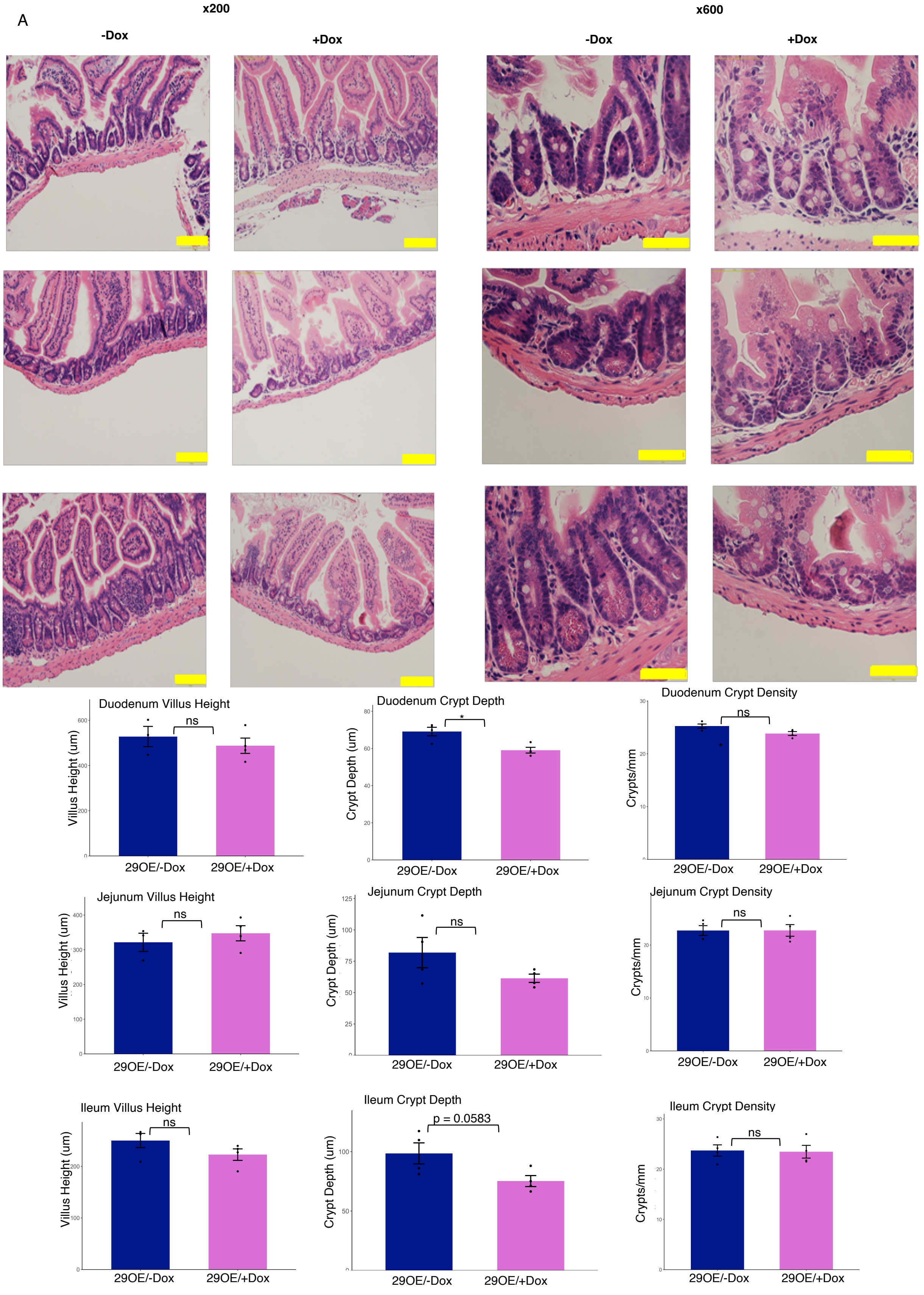

Supplemental Figure 3 - WT Histomorphometry

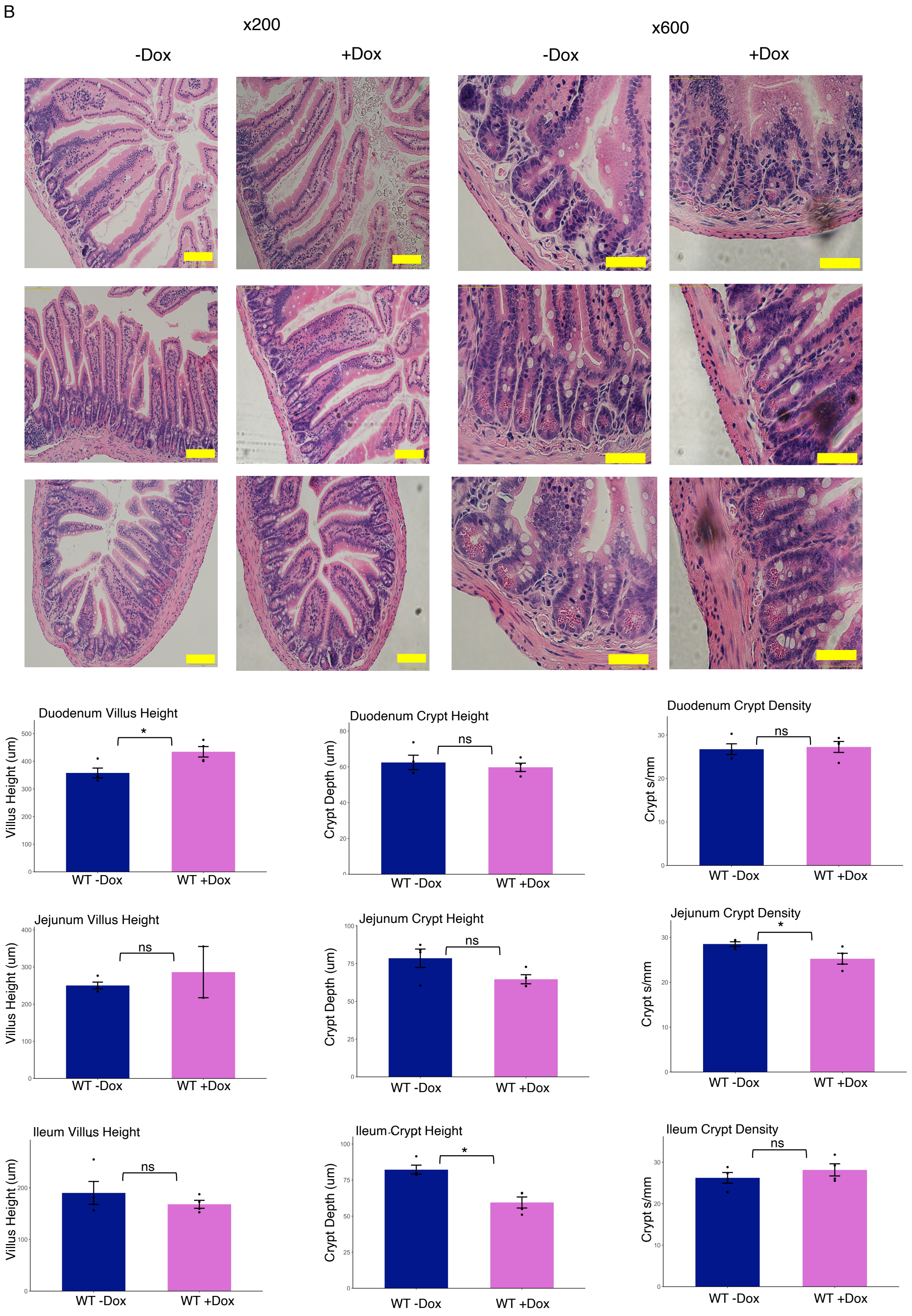

Supplemental Figure 3 – 29OE Second Round Histomorphometry

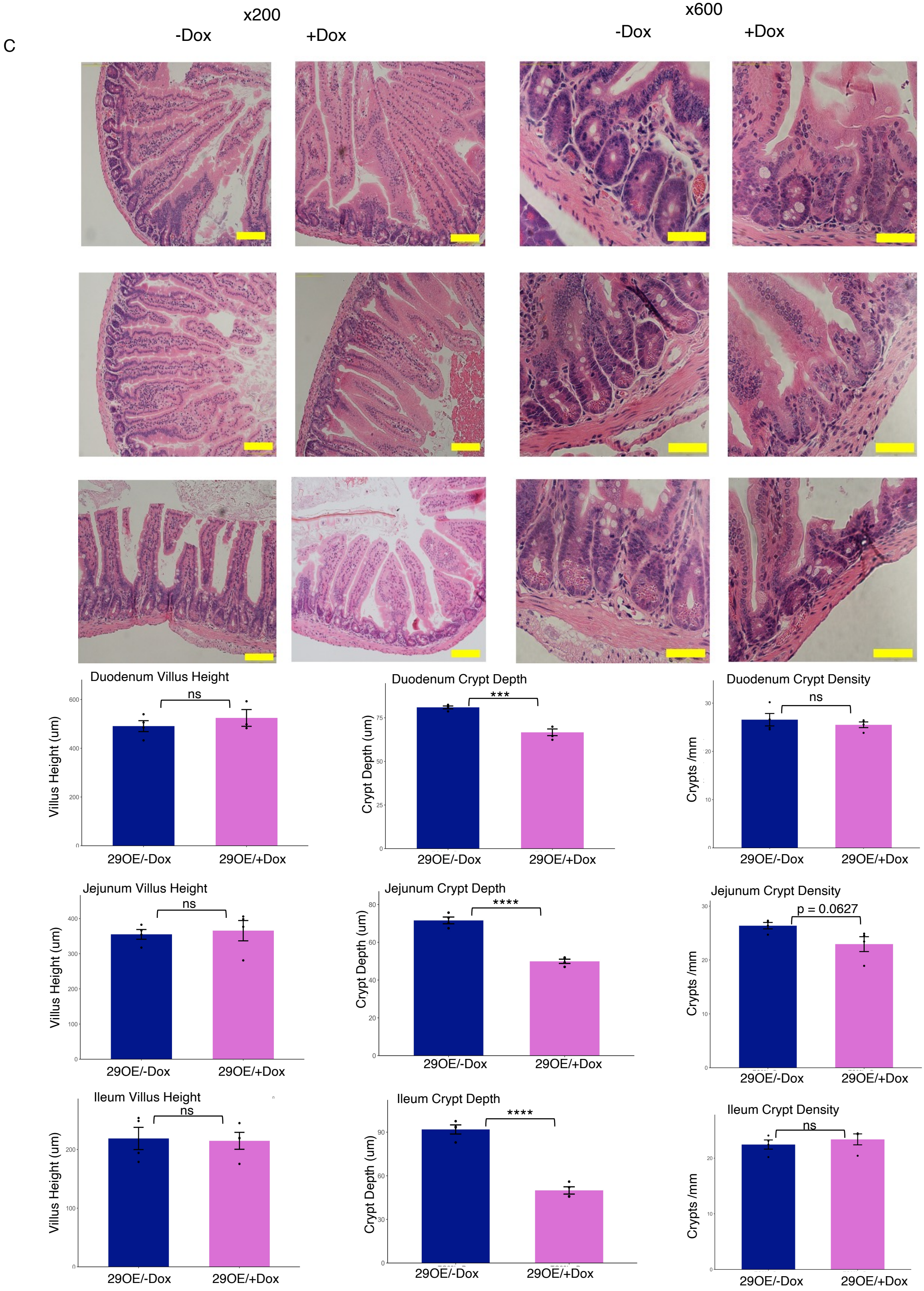

Supplemental Figure 3. Small intestinal architecture is unaffected by miR-29b overexpression. (A,B) Histomorphometry of 29OE/–Dox (n=4) and 29OE/+Dox (n=4) mice from initial (A) and repeated (C) experimental rounds, and of (B) wild-type (WT) mice without doxycycline treatment (WT/-Dox) (n=4) and with doxycycline treatment (WT/+Dox) (n=4). Villus height, crypt depth, and crypt density were measured for the proximal duodenum, mid-jejunum, and distal ileum. Representative brightfield H&E-stained images for villi and crypts are shown at x200 and x600 magnification, respectively. Yellow scale bars represent 100 µm at x200 magnification and 50 µm at x600 magnification. Dark blue represents the –doxycycline treatment and pink represents the +doxycycline treatment. Data presented as mean ± SEM (* $p < 0.05$, ** $p < 0.01$, *** $p < 0.001$, **** $p < 0.0001$; Student's t-test and Mann Whitney).

Supplemental Figure 4

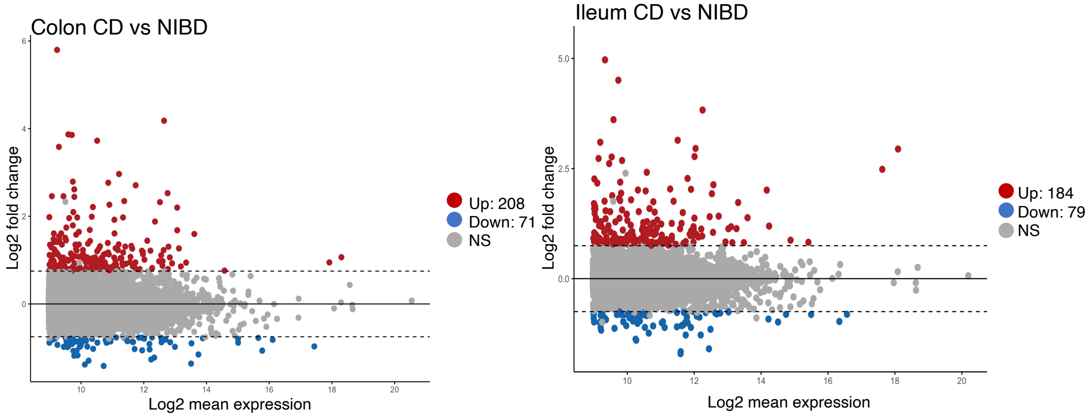

Supplemental Figure 4. MA plot showing genes that are significantly differentially expressed from RNA-seq data in pediatric CD vs NIBD patients (n=203) in the colon (left) and ileum (right) (baseMean > 500). Dashed lines represent the log2 fold-change of expression –0.75/+0.75 (horizontal). Up- or downregulated genes are colored red or blue, respectively, with an adjusted p-val < 0.05.

Supplemental Figure 5

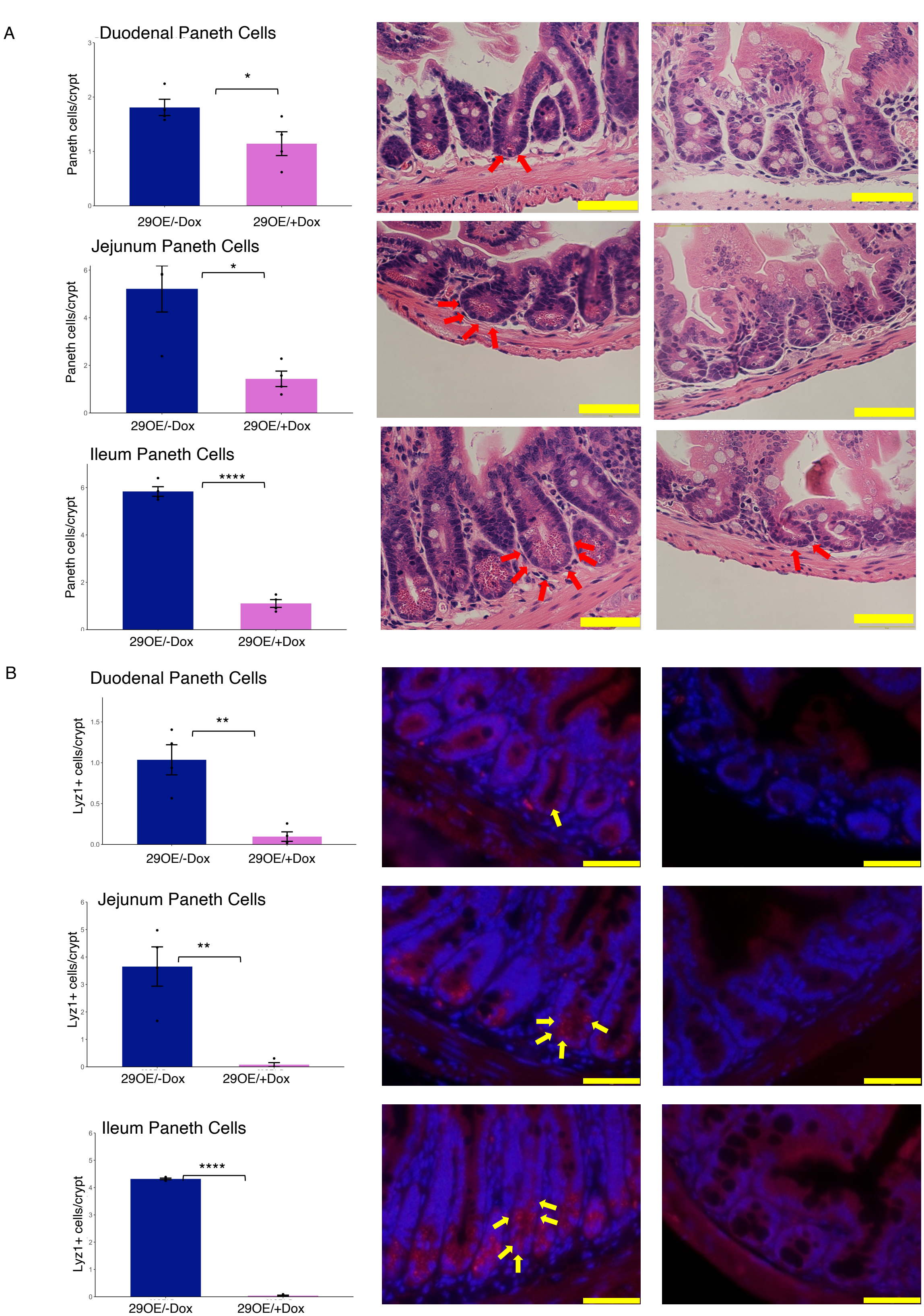

Supplemental Figure 5. Over-expression of miR-29b reproducibly reduces the number of Paneth cells. (A) Paneth cell counts per crypt of 29OE/-Dox (n=4) and 29OE/+Dox (n=4) mice for the proximal duodenum, mid-jejunum, and distal ileum from brightfield images H&E-stained tissue sections (x600). (B) Paneth cell counts per crypt of 29OE/-Dox (n=4) and 29OE/+Dox (n=4) mice for the proximal duodenum, mid-jejunum, and distal ileum from Lyz1 immunofluorescent (red) and DAPI fluorescent (blue) images (x600). Yellow scale bars measure 50 µm. Individual Paneth cells are indicated by red arrow bars. Dark blue represents the –doxycycline treatment and pink represents the +doxycycline treatment. Data presented as mean ± SEM for round one results (out of two total rounds) (* p< 0.05, ** p< 0.01,**** p< 0.0001; Student's t-test and Mann-Whitney).

Supplemental Figure 6

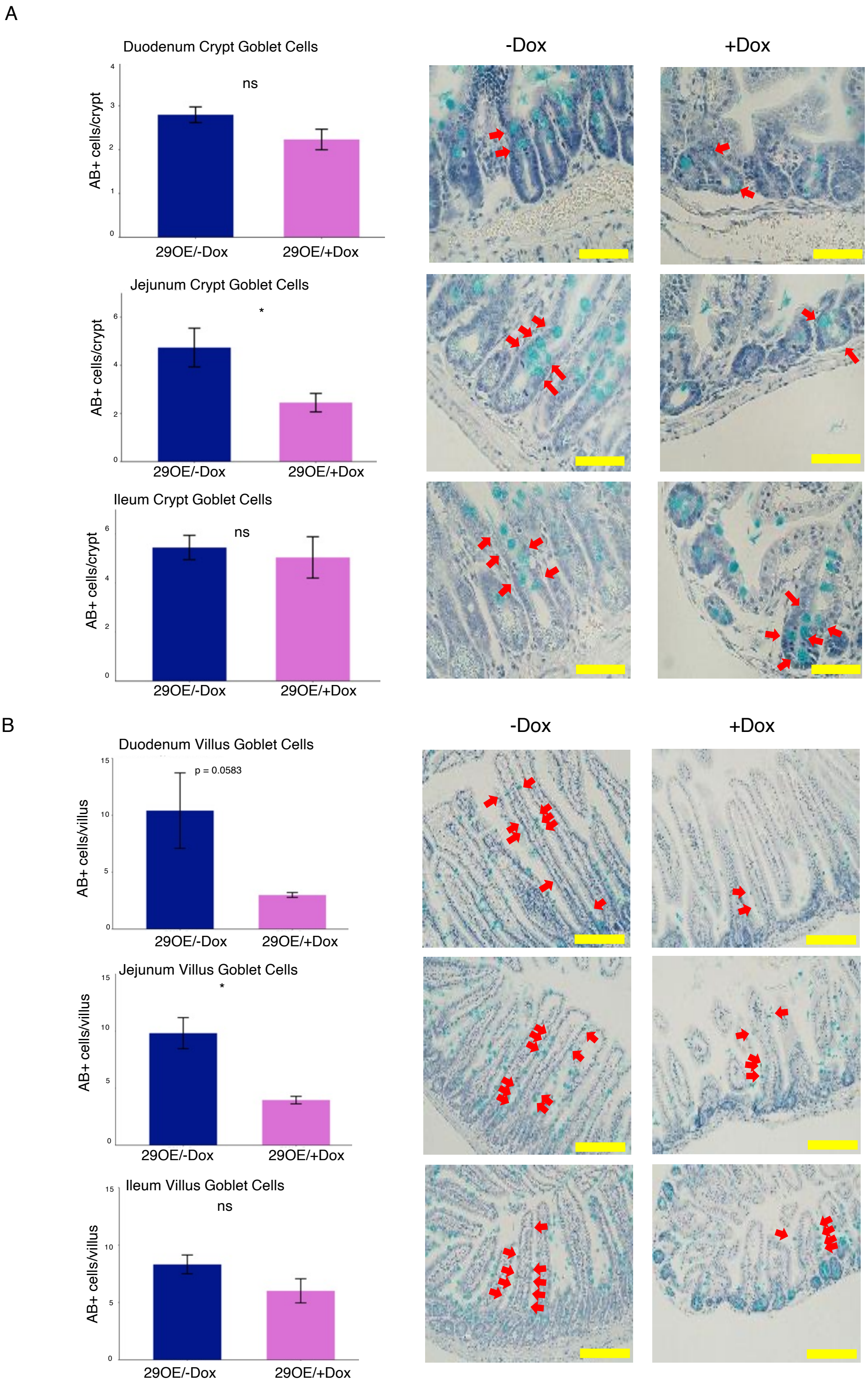

Supplemental Figure 6. Goblet cell counts per crypt (A) or per villus (B) from Alcian blue and eosin–stained tissue sections of 29OE/–Dox (n=4) and 29OE/+Dox in the proximal duodenum, mid-jejunum, and distal ileum (x600 images for crypts, x200 images for villi). Yellow scale bars represent 50 μm at x600 and 100 μm at x200. Individual Paneth and goblet cells are indicated by red arrows. Dark blue represents the –doxycycline treatment and pink represents the +doxycycline treatment. Data presented as mean ± SEM for round two results (out of two total rounds) (* $p < 0.05$, ** $p < 0.01$, **** $p < 0.0001$; Student's t-test and Mann-Whitney).

Supplemental Figure 7

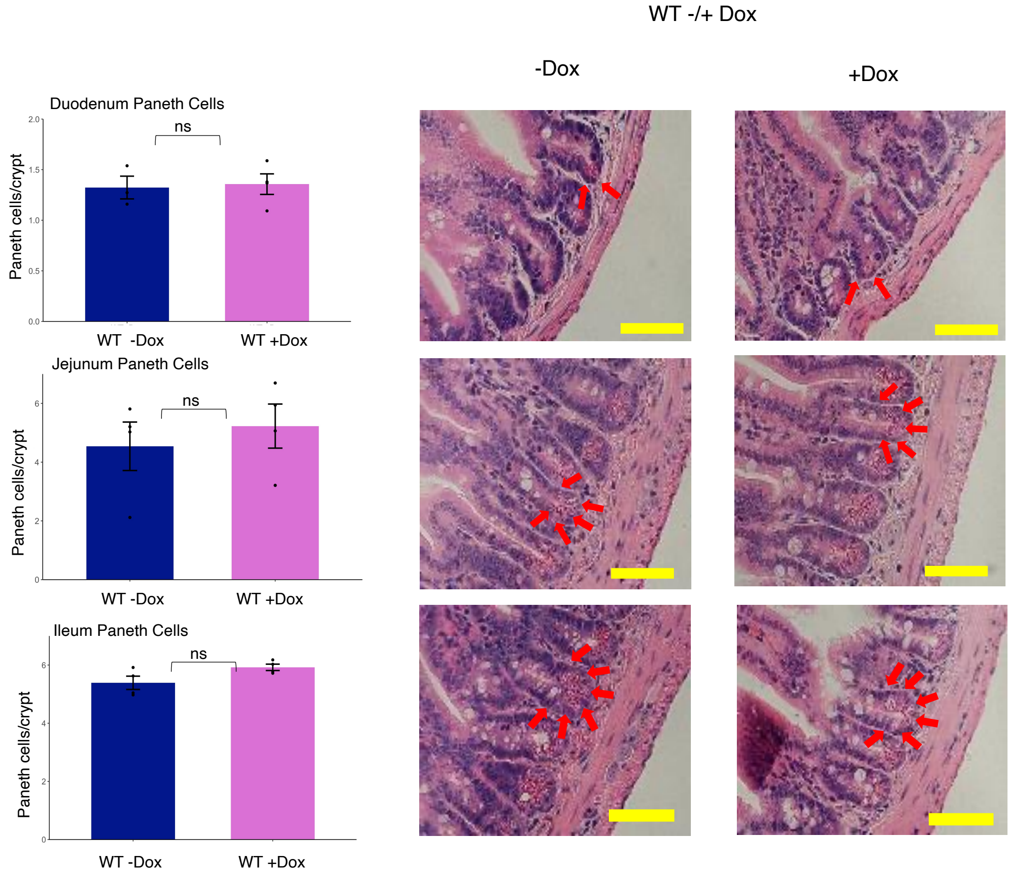

Supplemental Figure 7. Doxycycline treatment of wild-type mice does not affect the number of Paneth cells. (A) Paneth cell counts per crypt of WT/-Dox (n=4) and WT/+Dox (n=4) mice for the proximal duodenum, mid-jejunum, and distal ileum. Representative brightfield H&E-stained images at x600 magnification. Yellow scale bar measures 50 µm. Individual Paneth cells are indicated by red arrows. Dark blue represents the –doxycycline treatment and pink represents the +doxycycline treatment. Data presented as mean ± SEM (n.s.=not significant; Student's t-test).

Supplemental Table 1. Table of all clinical parameters evaluated for logistic regression analysis.

Clinical Parameters	Binary or Categorical	Colon CD	Ileum CD
Perianal Disease	Binary	24	21
Rectal or Sigmoid Involved?	Binary	35	34
Surgery with Anastomosis	Binary	27	21
Peri-anal Surgery	Binary	11	10
Temporary Ileostomy?	Binary	6	5
# of times therapy escalated (Average)	Categorical	1.32	1.27
Current Biologic?	Binary	40	33
Current IM?	Binary	39	35
Remission?	Binary	58	50
Sex	Binary	F = 27<br>M = 48	F = 22<br>M = 43
Diagnosis Age	Categorical	VEO = 2<br>Child = 36<br>Teen = 37	VEO = 3<br>Child = 31<br>Teen = 31
Family History of IBD	Binary	30	25
Locations	Categorical	L1 = 11<br>L2 = 11<br>L3 = 49<br>L4 = 4	L1 = 10<br>L2 = 8<br>L3 = 43<br>L4 = 4
Failed IM?	Binary	31	26
Type of Ileal Disease	Categorical	Modest Inflammation =15<br>Severe Inflammation = 41<br>Stricturing = 16<br>Penetrating = 3	Modest Inflammation =13<br>Severe Inflammation = 38<br>Stricturing =13<br>Penetrating = 1

Supplemental Table 2 Results from the multinomial logistic regression analysis with multiple testing correction (FDR) applied to the p-values. Two clinical parameters (Rectal/sigmoid involvement and Family History of IBD) had significant adjusted p-values with p< 0.1 for the colonic tissue samples. One clinical parameter (Surgery with anastomosis) had one microRNA with significant adjusted p-values with p< 0.1 for the ileal tissue samples

Colonic microRNA	Rectal or Sigmoid Involved
hsa-miR-21-5p	0.00508425
hsa-miR-21-5p_+_1	0.00508425
hsa-miR-21-3p	0.01109579
hsa-miR-31-5p	0.01416445
hsa-miR-26a-5p_-_1	0.05250646
hsa-let-7b-5p	0.07157595
hsa-miR-215_-_1	0.07157595

Colonic microRNA	Family History
hsa-miR-142-5p	0.0703179
hsa-miR-142-5p_+_1	0.0703179
hsa-miR-16-5p	0.0703179
hsa-miR-215_-_1	0.0703179
hsa-miR-29a-3p	0.0703179
hsa-miR-15a-5p	0.07101881
hsa-miR-142-3p_+_3	0.08506924
hsa-miR-451a	0.08506924

Ileal microRNA	Surgery with Anastomosis
hsa-miR-215_-_1	0.09907668

Supplemental Table 3. List of 16 genes that are significantly down-regulated in both the ileum of pediatric CD patients (relative to NIBD controls) (baseMean > 150, log2FC <-0.5, padj <0.05) and in IECs of 29OE/+Dox mice (relative to 29OE/-Dox controls) (baseMean > 150 , log2FC <-0.5 , padj<0.05).

Gene	Species/Tissue Located in
DSC2	Pediatric Colon, Pediatric Ileum, OE 29 Mouse
EPB41L4B	Pediatric Colon, Pediatric Ileum, OE 29 Mouse
EPCAM	Pediatric Colon, Pediatric Ileum, OE 29 Mouse
HSPA1B	Pediatric Colon, Pediatric Ileum, OE 29 Mouse
LGALS3	Pediatric Colon, Pediatric Ileum, OE 29 Mouse
PRR15	Pediatric Colon, Pediatric Ileum, OE 29 Mouse
ACSF2	Pediatric Ileum, OE 29 Mouse
DNASE1	Pediatric Ileum, OE 29 Mouse
ENPP3	Pediatric Ileum, OE 29 Mouse
FAM151A	Pediatric Ileum, OE 29 Mouse
NR1D2	Pediatric Ileum, OE 29 Mouse
PDZK1	Pediatric Ileum, OE 29 Mouse
PMP22	Pediatric Ileum, OE 29 Mouse
SLC52A3	Pediatric Ileum, OE 29 Mouse
SLC5A1	Pediatric Ileum, OE 29 Mouse
XPNPEP2	Pediatric Ileum, OE 29 Mouse
